# Co-benefits of reduced carbon and water footprints and enhanced carbon sequestration with integrated organic–inorganic fertilization and cover cropping in hilly citrus orchards

**DOI:** 10.3389/fpls.2026.1763629

**Published:** 2026-02-25

**Authors:** Wenwen Ning, Jian Zhao, Prakash Lakshmanan, Tieguang He, Shuai Wang, Yuanlong Ran, Pengjie Zhan, Zeyu Wang, Sili Ye, Yu Xiang, Yi Wen, Xiaojun Shi, Jingkun Zhao, Yuting Zhang

**Affiliations:** 1 College of Resources and Environment, Interdisciplinary Research Center for Agriculture Green Development in Yangtze River Basin, Southwest University, Chongqing, China; 2 College of Elementary Education, Chongqing Normal University, Chongqing, China; 3 Sugarcane Research Institute, Guangxi Academy of Agricultural Sciences, Nanning, China; 4 Agricultural Resources and Environmental Research Institute, Guangxi Academy of Agricultural Sciences/Guangxi Key Laboratory of Arable Land Conservation, Nanning, China; 5 Chongqing Agricultural Technology Extension Station, Chongqing, China; 6 Zhongxian Agricultural Science and Technology Extension Center, Chongqing, China

**Keywords:** carbon footprint, hilly citrus orchard, life cycle assessment, optimal nutrient management, water footprint

## Abstract

Citrus, a globally significant fruit crop, is predominantly cultivated on sloping land in China with a large amount of resource input and incurs high environmental costs. Current research often relies on general parameters and rarely simultaneously assesses carbon footprint (CF) and water footprint (WF) to reveal the synergistic effects in emission reduction strategies. To address knowledge gaps, we conducted a 2-year county-scale survey and a 3-year field experiment in Zhongxian County, Chongqing, China. We optimized five nutrient management schemes, chemical fertilizer (Che), chemical fertilizer + organic manure (Che+Org), chemical fertilizer + cover crops (Che+CC), chemical fertilizer + organic manure + cover crops (Che+Org+CC), and optimized management (OPT), and analyzed them using the life cycle assessment (LCA) framework. The results showed that OPT achieved dual benefits of high productivity and low carbon–water cost, with a CF reduction of 26.9%–64.6% and a WF reduction of 75.7%–92.1% compared with other treatments. Nitrogen fertilizer production and application were the primary CF sources, whereas cover crop integration markedly decreased WF. A significant positive correlation between CF and grey WF (*p* < 0.05) indicates that cover crops simultaneously mitigated carbon emissions and reduced nitrogen/phosphorus runoff. While achieving these environmental benefits, the citrus yield of the OPT was 33.57% higher than that of the Che, and the economic returns were 45.51% higher. This study demonstrates that in the sloping land environment, selectively combining organic fertilizers and cover crops can transform the contradiction between yield and the environment into a synergistic effect, thereby deepening the understanding of sustainable nutrient management. The research results show that the OPT system is a superior nutrient management strategy for sloping citrus orchards. The research results also provide reliable and specific evidence to support the optimization of the “organic substitution” policy and offer a feasible approach for low-carbon, high-efficiency citrus production in ecologically fragile regions.

## 1 Introduction

Citrus is the most widely grown and the most productive fruit crop worldwide, grown over 0.15 billion hectares and producing 1.37 billion tons of fruit annually (FAO, 2022). China is the largest citrus producer in the world, and the annual production has increased from 876 million tons in 1990 to almost 6,000 million tons in 2020 (FAO, 2022). This increase was achieved with large inputs of agricultural resources, expanding the cultivation to increasingly steep hill slopes, but with high environmental costs. For example, the average input rates of nitrogen (N), phosphorus (P), and potassium (K) fertilizer in China’s major citrus production areas were as high as 485, 198 (P_2_O_5_), and 254 (K_2_O) kg ha^−1^, respectively (Li et al., 2019). Moreover, the partial factor productivity (PFP) from applied N fertilizer was only 54.6 kg yield kg^−1^ N, which is approximately 30% of PFP realized in other citrus production countries such as USA, Brazil, and South Africa (Li et al., 2019). The excessive use of chemical fertilizers in citrus production in China not only raises production costs but also becomes an important source of greenhouse gas (GHG) emissions (Wu et al., 2021), therefore, implementing precise nutrient management based on crop requirements is of vital importance for reducing resource waste and environmental footprint (Kang et al., 2020). Studies have shown that the total carbon (C) emissions of typical citrus orchards in China can reach 7.1–16.5 tons of carbon dioxide equivalent per hectare, with over 80% of this attributed to the use of fertilizers (Chen et al., 2020; Yan et al., 2016). Moreover, China’s carbon emissions are significantly higher than those of the United States and Brazil (Bell and Horvath, 2020; Veiga et al., 2024). Therefore, optimizing citrus orchard nutrient management to reduce its CF is critical for sustainable citrus production.

To address this challenge, the Ministry of Agriculture and Rural Affairs of the People’s Republic of China introduced the “Action Plan for organic substitution of chemical fertilizers (OSCF) for Fruits, Vegetables and Tea” in 2017. This policy aims to reduce the use of chemical fertilizer and promote the judicious utilization of livestock manure and cover crops to mitigate the global warming potential (Lal, 2015; Tang et al., 2019). This ecologically favorable outcome is mainly attributed to the following desirable features of organic inputs which include (i) reduced reactive N (Nr) losses and GHG emissions by substituting an appropriate amount of chemical fertilizer (Xia et al., 2017); (ii) increased soil organic C (SOC) stock (Seitz et al., 2023; Iqbal et al., 2022); (iii) improved soil physicochemical and biological properties, which in turn reduce soil erosion and increase soil nutrient retention capacity (Xie et al., 2014; Li et al., 2019); and (iv) promoting of carbon sequestration through improved crop growth (Fang et al., 2022; Zanotelli et al., 2015). In orchard ecosystems located in hilly areas, these benefits accrued from cover crops can be remarkable. Our previous study reported that, compared with bare fallow, cover crops grown in interrow spaces in citrus orchards can significantly reduce soil, N, and P runoffs by as much as 70.5%, 53.4%, and 56.9%, respectively (Liu et al., 2021a) and enhance soil C stock by 2.0 Mg ha^−1^ year^−1^ on a global scale (Hu et al., 2022). The Inter-governmental Panel on Climate Change (IPCC) has also recommended farmers to adopt these integrated and organic production practices in order to make agriculture sustainable and mitigate climate change (IPCC, 2014). In Europe, fruit production is considered as a low environmental cost agricultural sector, or even as a C sink ecosystem, which was accomplished through efficient organic fertilization and cover crop management (Zanotelli et al., 2015; Chamizoa et al., 2017). However, in the specific hilly terrain, climate, and planting patterns in China, the combined management strategy of organic fertilizers and cover crops, and its quantitative impact on the comprehensive environmental benefits of the citrus production system, especially the synergistic reduction effect on CF and WF, is still lacking a clear elaboration based on localized empirical evidence.

Currently, CF and WF are commonly used to estimate the environmental burdens arising from fruit production (Finkbeiner, 2009). The CF has been widely used to estimate the agricultural contribution to global climate change and to explore mitigation measures for GHG emissions (Rebolledo-Leiva et al., 2017). The WF concept was introduced as an integration of the total evapotranspiration of rainwater (defined as green water), evapotranspiration of surface water/groundwater (blue water, i.e., water stored in rivers, lakes, or shallow groundwater layers), and the volume of freshwater required to regulate the pollutant loads to maximum acceptable levels (grey water) (Aldaya et al., 2011). The WF, especially grey WF, is directly linked with the nutrient losses and water quality (i.e., NO_3_^−^ and PO_4_^−^ concentration) and positively related with CF (Hogeboom, 2020; Zhang et al., 2018). Although previous studies have separately evaluated the total CF of citrus production in Fujian Province of China (Chen et al., 2020) and Hubei Province (Yan et al., 2016) using farm surveys and field experiment data, these evaluations have significant limitations. Firstly, in China, citrus is mainly grown in low mountains and hills, and the indirect N_2_O emissions caused by nutrient loss in runoff were not taken into account in these studies, which led to seriously underestimated CF estimates (Graham et al., 2017). Secondly, the GHG calculation parameters used in previous studies were all taken from the Intergovernmental Panel on Climate Change or non-farmland studies (Chen et al., 2020; Yan et al., 2016), and these parameters did not reflect the local citrus production conditions. Finally, the existing studies mostly conducted isolated analyses of CF and WF, failing to reveal the synergistic responses of the two to the same management measures, which is crucial for formulating sustainable orchard management.

To fill these knowledge gaps, in this study, we conducted a 2-year county-scale survey and a 3-year nutrient management field experiment of citrus in the upper reaches of Yangtze River valley of China and evaluated CFs and WFs of citrus production under different nutrient management strategies. The aims of this study are (i) to assess the environmental efficacy of the OSCF policy proposed by the Chinese government on citrus production and (ii) to optimize a citrus nutrient management strategy to reduce environmental footprints, including GHG emission from citrus production. The new knowledge arising from this study could provide a reference and theoretical support for developing policies for sustainable orchard management and citrus production.

## 2 Materials and methods

### 2.1 Study site

**Figure 1:**
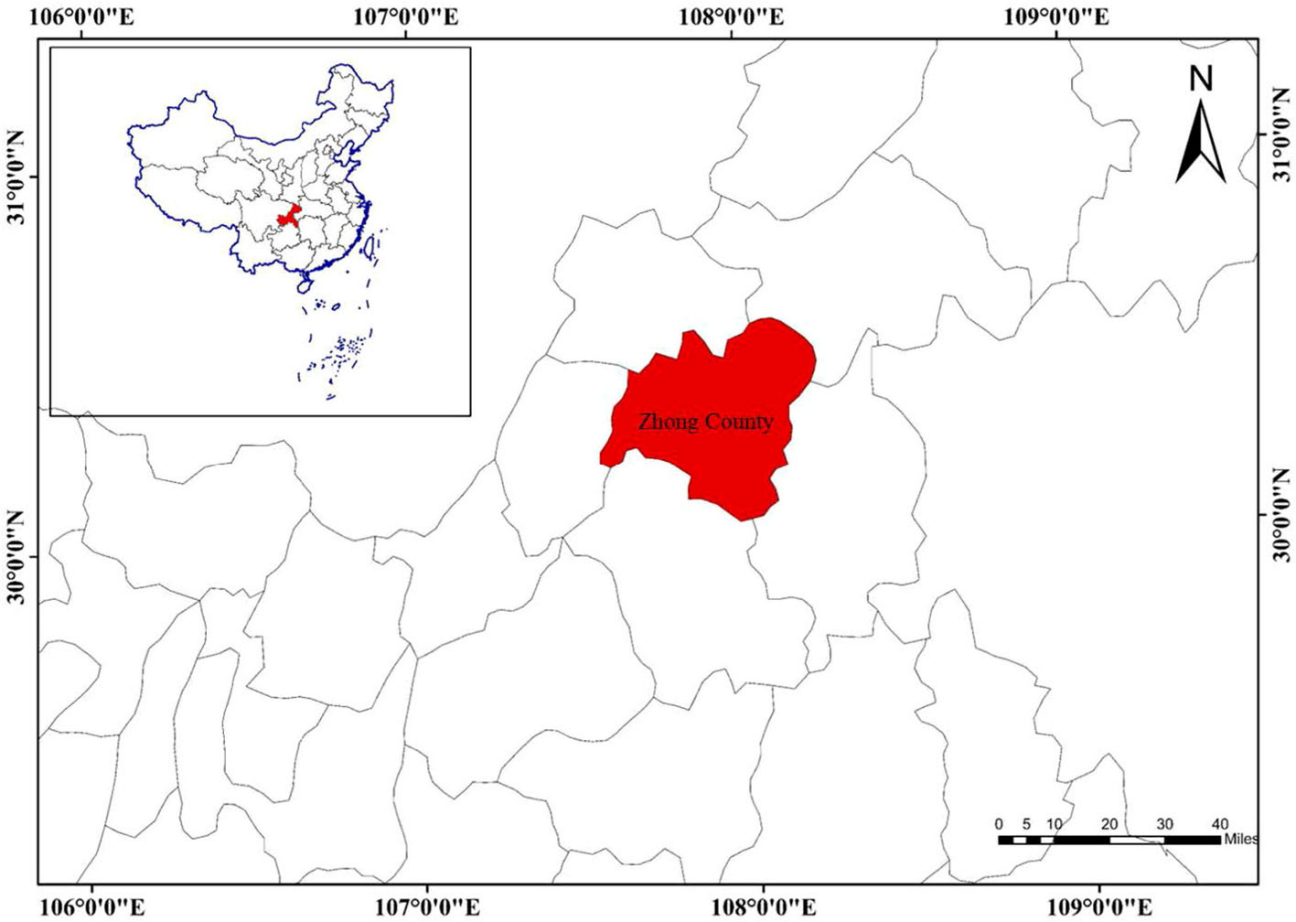
Location of the study site in China.

The study site was in Zhong County of Chongqing, China, which is located in the Three Gorges Reservoir area of the upper reaches of Yangtze River valley (107°32′~108°18′E, 30°03′~30°53′N) (**Figure 1**). This site has a subtropical monsoon climate, with a mean annual precipitation of 1,200 mm and a mean annual temperature of 18.2°C, which are particularly suitable for citrus cultivation. The region is the main citrus-producing area of China, and Zhong County is the core citrus production demonstration area. In 2020, the citrus plantation area in Zhong County was 23,600 ha, accounting for approximately 31% of the total arable land and 82.1% citrus planted on hilly areas in this county. Citrus annual production averaged 338 million tons with the output value of ~30 billion yuan RMB, thus contributing 6.15% to the total GDP of this country.

Various citrus varieties are cultivated in the study region, which include the early-maturing Daya mandarin (*Citrus reticulata ‘Daya’*) (DM), Harumi mandarin (*Citrus reticulata ‘Harumi’*) (HM), Lane Late Navel Orange (*Citrus sinensis (L.) Osb.*) (LLN), and Orah mandarin (*Citrus reticulate ‘Orah’*) (OM) and the late-maturing Ehime mandarin (*Citrus reticulata ‘Ehime’*) (EM) and Shatangju mandarin (Citrus reticulata ‘Shatangju’) (StjM) varieties. They were all used in this study. From 2017 to 2021, the Zhong county government encouraged farmers to implement different forms OSCF by offering free organic fertilizers and cover crop seeds, and thus various nutrient management strategies in citrus production can be found in this area.

### 2.2 System boundary

**Figure 2:**
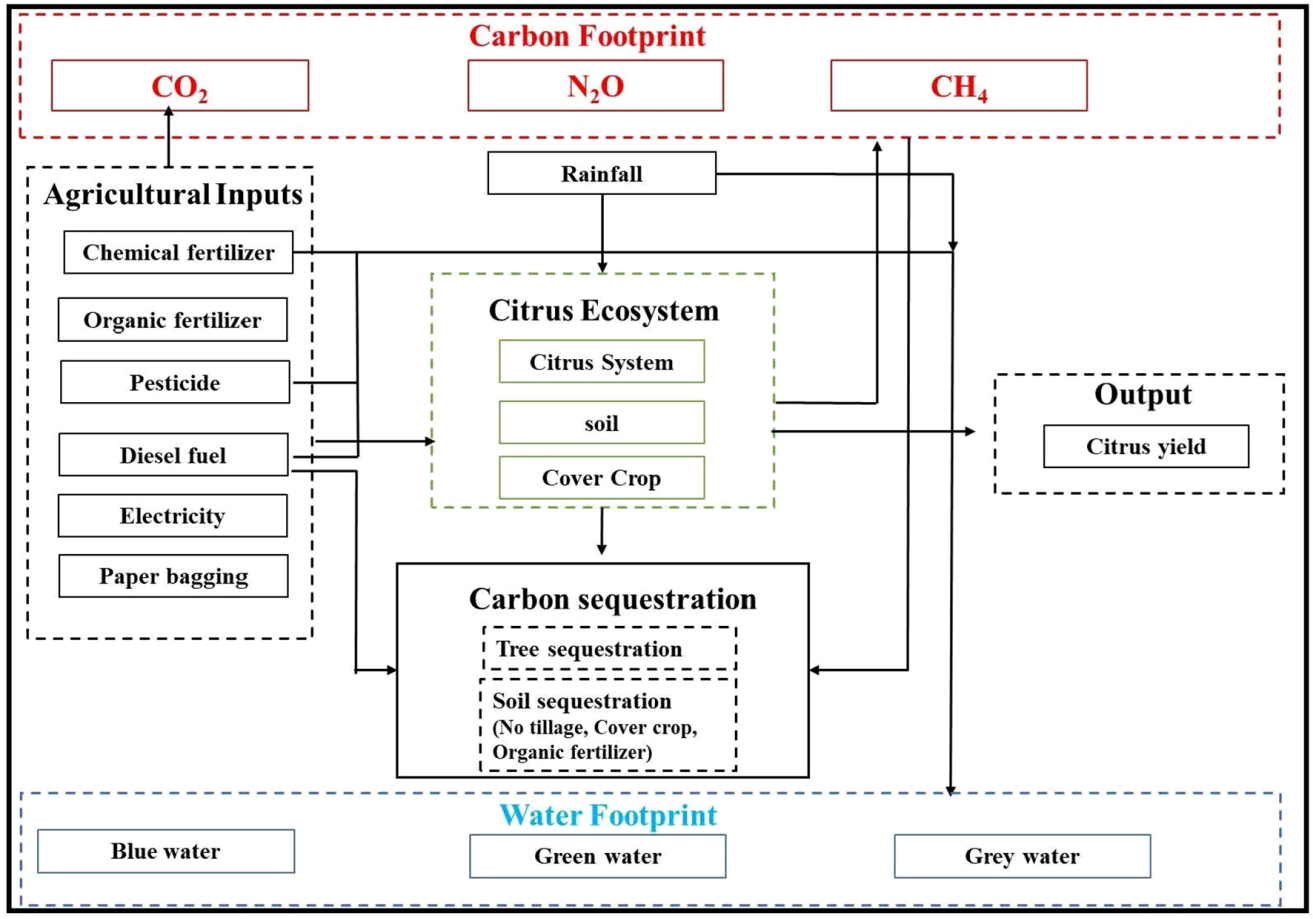
System boundary of the citrus production in this study.

In this study, the CF and WF of different citrus production systems were evaluated with a cradle-to-farm gate life cycle assessment methodology. The system boundary included agricultural material production and citrus production sections (**Figure 2**), and both GHG emissions (GE) and C sequestration were also considered. The GHG emissions included CO_2_ (from input production and energy use), direct and indirect N_2_O emissions (ammonia (NH_3_), and the N losses in runoff and leaching), and CH_4_ emissions from organic amendments. Carbon sequestration in soil and perennial biomass (from organic inputs, cover crops, no-tillage, and tree growth) was included as a negative emission. Excluded processes were post-farmgate activities such as fruit transport, processing, and distribution, as the study focuses on comparing on-farm management practices. Capital goods and infrastructure (e.g., machinery, irrigation systems, buildings) were also excluded, as their contribution to the annual footprint of perennial cropping systems is typically negligible compared with operational inputs.

**Figure 3:**
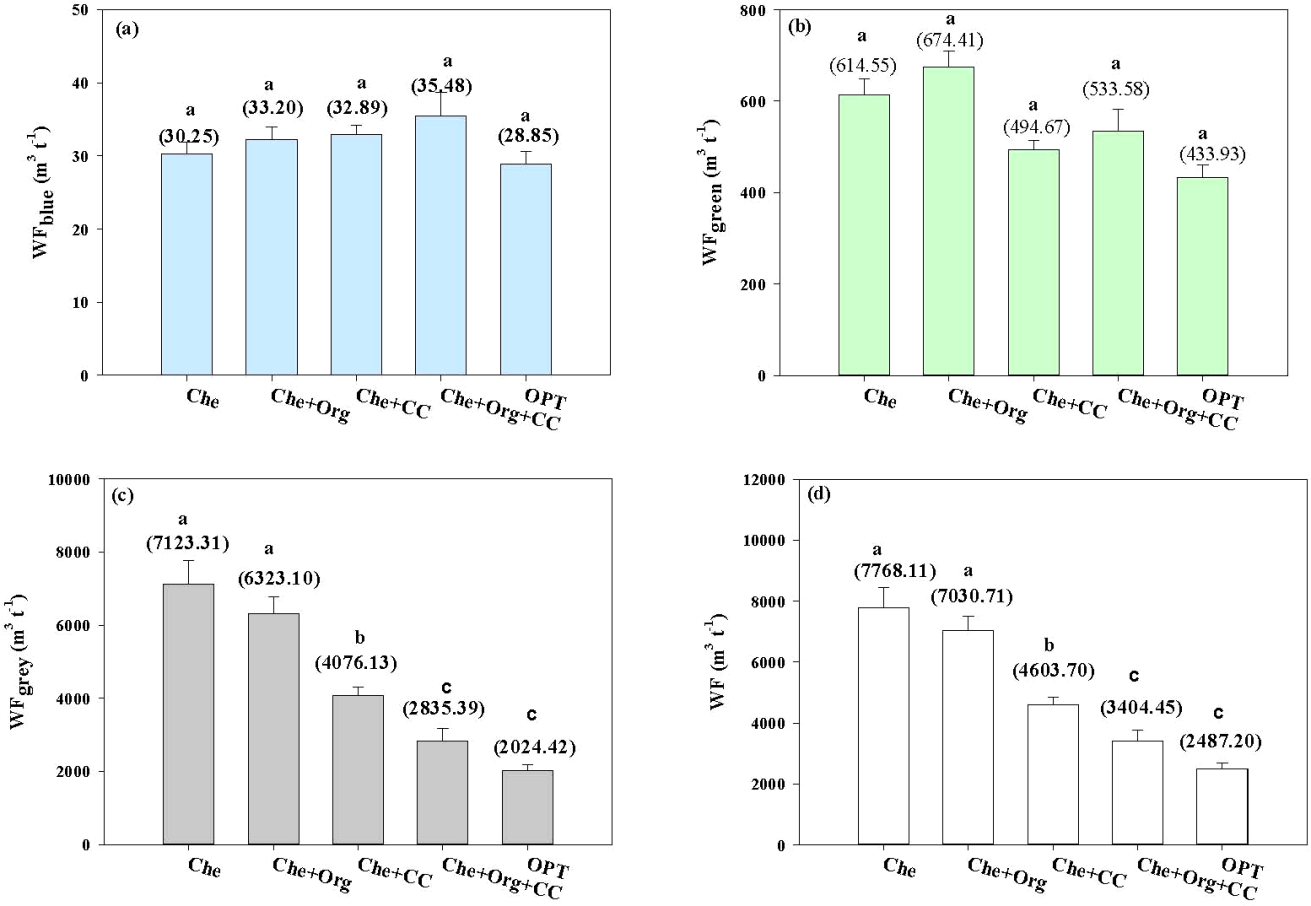
Effects of different nutrient management on blue water footprint **(a)**, green water footprint **(b)**, grey water footprint **(c)**, and total water footprint **(d)** of citrus orchards. WF_blue_ represents the fresh surface water or groundwater that either evaporates or incorporated into the citrus product. The WFgreen refers to the precipitation that is stored in or stays in the soil, WFgrey is defined as the volume of freshwater that is required to assimilate the load of pollutants based on natural background concentrations and existing ambient water quality standards.WF means the total water footprint. Che, Che+ Org, Che+ CC, Che+ Org+ CC, and OPT refer to chemical fertilizer only, chemical fertilizer plus organic fertilizer, chemical fertilizer plus cover crop, and chemical fertilizer plus combined organic material inputs and optimal nutrient management. Different letters after peer data indicate that the zone groups are significantly different at the 0.05 level, and numbers in brackets indicate water consumption.

In the system boundary of citrus production, the WF was estimated based on the consumption of rainfall (green WF) and irrigation water (blue WF), and the freshwater required to dilute contaminated water by total N and P losses from fertilizer (grey WF). The specific impact results of each management measure on each component of water footprint are shown in **Figure 3**.

### 2.3 Data collection and nutrient management strategies

The data sources included a 2-year county-scale survey and a 3-year citrus nutrient management experiment. The survey was conducted systematically through face-to-face meeting supported by a comprehensive questionnaire encompassing all aspects of citrus production in 2020 and 2021, covering 273 smallholder or large-scale citrus agribusinesses and 48% townships in Zhong County. All of these in-house surveys were conducted by professional research staff to ensure that the survey samples are representative. The questionnaire collected information on citrus management, including fertilizer application, pesticide application, diesel fuel and electricity consumption, fruit bagging, cover crop and tillage management, irrigation, citrus variety, yields, citrus tree annual growth biomass, farmer’s cost, and economic outcomes.

Following the different OSCF Policy strategies carried out in the orchard farms, the survey data was divided into four nutrient management strategies, namely, chemical fertilizer only (Che), chemical fertilizer plus organic fertilizer (Che+Org), chemical fertilizer plus cover cropping management (Che+CC), and chemical fertilizer plus combined organic material inputs (Che+Org+CC). Optimal fertilization plus cover crop (OPT), for which data were obtained from a 3-year orchard field experiment, was also included for analysis. The OPT mode aims to integrate the core principles of OSCF: reduction of chemical fertilizer use, precise application of organic fertilizers, and management of cover crop systems. In the OPT treatment, the total nitrogen input is 300 kg N per hectare, of which 220 kg N per hectare is chemical nitrogen fertilizer (accounting for 73.3%) and 80 kg N per hectare is organic nitrogen fertilizer (accounting for 26.7%), with the ratio of inorganic nitrogen to organic nitrogen being approximately 2.75:1. Compared with the standard OSCF policy recommended dosage, the physical input of organic fertilizer in the OPT treatment is numerically lower. This design is based on optimization principles rather than simply following the policy. Our field experiments aim to determine, while maintaining yield and enhancing carbon sequestration capacity, the minimum effective organic input quantity required to maximize fertilizer reduction. Therefore, OPT represents a refinement and local calibration of the OSCF policy, shifting the focus from the general recommended dosage of organic substitution to a precise nutrient-integrated management strategy. This experiment was carried out in a typical citrus-growing hilly orchard in Zhong country and aimed to define and recommend an optimal nutrient management strategy to farmers, which include the criteria such as chemical fertilizer reduction, organic fertilizer application, and cover crop management. The agricultural inputs and outputs data from survey and experiment are shown in **Table 1**.

**Table 1:** Agricultural inputs and outputs in a typical hill slope citrus production system obtained from a survey involving 273 farms and a three-year nutrient management experiment to optimize nutrient supply.

Variable	Che	Che + Org	Che + CC	Che + Org +CC	OPT
Number of Researchers involved	40	38	70	125	–
Input					
Total fertilizer (kg ha^-1^)					
N	301.80±28.82^ab^	353.46±18.57^ab^	406.16±20.22^a^	404.91±15.89^a^	300.00±0.00^b^
P_2_O_5_	171.80±18.40 ^a^	164.89±12.27 ^a^	228.77±15.68 ^a^	202.06±7.85 ^a^	167.25±0.00 ^a^
K_2_O	262.18±19.35 ^a^	286.10±19.54 ^a^	381.03±18.11 ^a^	358.56±16.45 ^a^	309.00±0.00 ^a^
Chemical fertilizer (kg ha^-1^)					
N	301.80±28.82 ^ab^	253.96±16.19 ^ab^	406.16±20.22 ^a^	300.27±13.69 ^ab^	220.00±0.00 ^b^
P_2_O_5_	171.80±18.40 ^ab^	114.93±10.61 ^b^	228.77±15.68 ^a^	150.64±6.93 ^ab^	137.25±0.00 ^b^
K_2_O	262.18±19.35 ^a^	233.96±19.79 ^b^	381.03±18.11 ^a^	307.20±14.90 ^ab^	279.00±0.00 ^ab^
Organic fertilizer (kg ha^-1^)					
N	–	99.50±13.63 ^a^	–	104.64±7.47 ^a^	80.00±0.00 ^a^
P_2_O_5_	–	49.96±6.82 ^a^	–	51.42±3.72 ^a^	40.00±0.00 ^a^
K_2_O	–	52.13±7.13 ^a^	–	51.36+3.73 ^a^	40.00±0.00 ^a^
Organic fertilizer (kg ha^-1^)	–	5502.00±1130.42^a^	–	3118.62±910.32^b^	4000±0.00^ab^
Pesticide (kg ha^-1^)	6.75±0.35 ^a^	7.50±0.32 ^a^	7.32±0.20 ^a^	6.70±0.25 ^a^	7.05±0.00 ^a^
Fuel consumption (L ha^-1^)	10.73±3.02 ^b^	26.63±6.05 ^ab^	8.23±2.82 ^b^	15.65±4.37 ^b^	17.4±0.00 ^a^
Electricity (kwh ha^-1^)	187.48±17.36 ^ab^	132.72±20.58 ^b^	249.64±0.00 ^a^	189.03±9.54 ^ab^	246.30±0.00 ^a^
Paper bags (kg ha^-1^)	8.42±5.53 ^a^	12.43±4.64 ^a^	10.77±4.01 ^a^	13.25±4.42 ^a^	–
Ground Management	Clean tillage	Clean tillage	Cover cropping	Cover cropping	Cover cropping
Cover crop biomass (t ha^-1^)	0	0	22.5	22.5	22.5
Tillage Management	No inter-row tillage	No inter-row tillage	No inter-row tillage	No inter-row tillage	No inter-row tillage
Output					
Yield (t ha^-1^)	12.450±0.78 ^a^	11.13±0.57 ^a^	16.94±0.99 ^a^	17.40±0.72 ^a^	16.63±1.00 ^a^
Economic benefit (×10^4^ yuan ha^-1^)	12.48±1.05 ^b^	11.82±1.32 ^b^	22.61±3.63 ^a^	19.20±1.68 ^a^	18.16±1.20 ^a^

Che, Che+ Org, Che+ CC, Che+ Org+ CC and OPT refer to chemical fertilizer only, chemical fertilizer plus organic fertilizer, chemical fertilizer plus cover crop, and chemical fertilizer plus combined organic material inputs, and optimal nutrient management, respectively. Different letters after each data indicate that the zone groups are significantly different at the 0.05 level.

### 2.4 Methodology

#### 2.4.1 GHG emissions

The CO_2_ emissions from citrus production is determined according to the emission factors of agricultural inputs, including the production of chemical fertilizers, organic fertilizers, and pesticides, diesel fuel consumption, electricity for irrigation, and fruit bagging (**Table 2**). The total CO_2_ emissions were calculated using the following Equation 1:

**Table 2:** Greenhouse gas emission factors of different sources associated with citrus production used for the estimation in this study.

Emission source	unit	Emission factor or scaling factor	References
Chemical N fertilizer	Kg N	8.3 kg CO_2_eq unit^-1^	Zhang et al. (2013)
Chemical P_2_O_5_ fertilizer	Kg P_2_O_5_	2.33 kg CO_2_eq unit^-1^	Chen et al. (2015)
Chemical K_2_O fertilizer	Kg K_2_O	0.66 kg CO_2_ eq unit^-1^	Chen et al. (2015)
Organic fertilizer	Kg	0.2 kg CO_2_ eq unit^-1^	Li et al. (2016)
Pesticide	Kg	19.1 kg CO_2_ eq unit^-1^	Clark et al. (2016)
Diesel fuel	Kg	3.75 kg CO_2_ eq unit^-1^	Pishgar-Komleh et al. (2013)
Electricity	KWh	0.75 kg CO_2_ eq unit^-1^	Yue (2013)
Paper bags	Kg	1.54 kg CO_2_ eq unit^-1^	Chen and Qiu (2014)
N_2_O emission from chemical N fertilizer	Kg	1.01%	Gu et al. (2019)
NH_3_ emission from chemical N fertilizer	Kg	13.38%	Kang et al. (2022)
N runoff and leaching from chemical N fertilizer	Kg	46.22% in clean tillage management, and 26.11% in cover crop management	Liu et al. (2021a)
N_2_O emission from organic fertilizer	Kg	0.6%	Zhang et al. (2017)
NH_3_ emission from organic fertilizer	Kg	29.30%	Zhang et al. (2017)
CH_4_ emission from organic fertilizer	Kg	0.2%	Zhang et al. (2017)

(1) $$GE_{CO_{2}}=\sum (I_{i}\times EF_{i})$$

where *GE_CO_2__* represents the total CO_2_ emissions induced by the *i*th type of agricultural annual input, I_i_ is the amount of the *i*th type of the CO_2_ source, and EF_i_ is the emission factor of the *i*th type of the CO_2_ source (**Table 2**). The results are expressed in units of tons of CO_2_ equivalents (CO_2_ eq).

The direct and indirect N_2_O emissions from the application of chemical N fertilizer and organic fertilizers were also estimated, using the following equation:

(2) $$GE_{Che-N_{2}O}=Che_{N}\times (EF_{Che-N_{2}O}+EF_{Che-NH_{3}}\times 0.01+EF_{Che-RL}\times 0.0075)\times 265\times 44/28$$

(3) $$GE_{Org-N_{2}O}=Org_{N}\times (EF_{Org-N_{2}O}+EF_{Org-NH_{3}}\times 0.01)\times 265\times 44/28$$

(4) $$GE_{N_{2}O}=GE_{Che-N_{2}O}+GE_{Org-N_{2}O}$$

where *GE_Che-N_2_O_*, *GE_Oorg-N_2_O_*, and *GE_N_2_O_* represent the total N_2_O emissions from chemical N fertilizer application, organic fertilizer application, and both chemical N fertilizer and organic fertilizer application, respectively; *Che_N_* and *Org_N_* are the annual application amounts of N from chemical fertilizer and organic fertilizer, respectively; *EF_Che-N_2_O_*, *EF_Che-NH_3__*, and *EF_Che-RL_* represent the emission factors of N_2_O, NH_3_, and N lost (by runoff and leaching) caused by chemical N fertilizer application, respectively (**Table 2**); *EF_Org-N_2_O_*, and *EF_Org-NH_3__* are the emission factors of N_2_O and NH_3_ caused by organic fertilizer application, respectively (**Table 2**); 0.01 and 0.0075 are the conversion coefficients of NH_3_ volatilization and N lost (by runoff and leaching) compared with N_2_O equivalents, respectively (IPCC, 2006); 44/28 is the molecular conversion factor of N_2_ to N_2_O; and 265 is the global warming potential of N_2_O compared with CO_2_ equivalents (IPCC, 2014).

Different from previous studies (Chen et al., 2020; Yan et al., 2016), the present study updated some emission factors according to the newly available data from peer-reviewed orchard articles and local experiments. For example, the N_2_O emission factor representing chemical N fertilization-induced emission was corrected by referring the global average N_2_O loss in subtropical orchard systems (Gu et al., 2019) (**Table 2**). Data from a local experiment (same soil type and climatic conditions) were adopted to correct the NH_3_ emission factor representing chemical N fertilization-induced emission (Kang et al., 2022) (**Table 2**). Also, most importantly, the N runoff and leaching factors under both conventional clean tillage and cover cropping management were adopted (**Table 2**) according to our previous research carried out in the same study region (Liu et al., 2021b).

The direct CH_4_ emissions from organic fertilizer application were also evaluated by the following equation:

(5) $$GE_{CH_{4}}=Org_{C}\times 0.2\%\times 28\times 16/12$$

where *GE_CH_4__* represents the total CH_4_ emissions caused by application of organic fertilizer; Org_C_ is the total carbon input by organic fertilization; 0.2% is the CH_4_ emission factor by organic fertilization (**Table 2**) (Wan et al., 2014); 16/12 is the molecular conversion factor of C to CH_4_; and 28 is the global warming potential of CH_4_ compared with CO_2_ equivalents (IPCC, 2014).

#### 2.4.2 Carbon sequestrations

The C sequestration is considered as C sink to mitigate GHG emissions and was calculated by the following equation:

(6) $$CS=(T_{CS}+Org_{CS}+CC_{CS}+NT_{CS})\times 44/12$$

where CS indicates the total amount of C sequestered in the citrus ecosystem and TC indicates the C assimilated by citrus tree photosynthesis, which is approximately 0.27 t C ha^−1^ year^−1^ as estimated by Wu (2008). *Org_CS_* represents the soil organic C sequestration rate caused by organic fertilization. According to previous research on global orchards (**Table 3**) (Aguilera et al., 2013), the conversion coefficient of the C source from organic fertilizer to SOC is 37.46%. The average C content of organic fertilizers used in this study area was 45.0%. *CC_CS_* represents the conversion coefficient of the C source from cover crop, which was 34.0% (**Table 3**) (Yang et al., 2020). In the present study, the average moisture content of cover crop was 82.1%, and dry biomass C content was 42.0%. *NT_CS_* represents soil organic C sequestration by non-tillage management, and it was 0.25 t C ha^−1^ year^−1^ (**Table 3**) (Lu et al., 2009). The 44/12 is the molecular conversion factor of C to CO_2_.

**Table 3:** Carbon sequestration factors of different sources associated with citrus production used for the estimation in this study.

Sequestration source	Sequestration factor or scaling factor	References
Organic fertilization	37.46%	Aguilera et al. (2013)
Citrus tree photosynthesis	0.27 t C ha^-1^yr^-1^	Wu (2008)
Cover cropping	34%	Yang et al. (2020)
No-tillage	0.25 t C ha^-1^yr^-1^	Lu et al. (2009)

#### 2.4.3 Carbon footprint calculation

The net GHG emission (NGE) was determined in terms of GE_CO_2__, GE_N_2_O_, GE_CH_4__, and CS using the following equations:

(7) $$NGE=GE_{CO_{2}}+GE_{N_{2}O}+GE_{CH_{4}}-CS$$

The farm CF (FCF, t CO_2_ eq ha^−1^) and product CF (PCF, t CO_2_ eq t^−1^), expressed in terms of NEG per unit orchard area and per unit of fresh citrus yield, respectively, were calculated using the following equations:

(8) FCF=NGEArea

(9) PCF=NGEYield

Carbon efficiency (yuan RMB t^−1^ CO_2_ eq), defined as economic benefit (total income minus costs, RMB ha^−1^, **Table 1**) earned by unit FCF in citrus production, was also calculated by the following equation:

(10) Carbon Efficiency=Economic BenifitFCF

### 2.5 Water footprint calculation

This study adopted a volumetric approach for quantification of WF of citrus production (PWF, m^3^ t^−1^), including those of green WF (WF_green_), blue WF (WF_blue_), and grey WF (WF_grey_). WF_green_ and WF_blue_ represent the consumption of water use (CWU, m^3^ ha^−1^) for citrus production and can be calculated as the following:

(11) WFgreen=CWUgreenYield=10×ETgreenYield

(12) $$ET_{green}=min(ET_{c},\;P_{eff})$$

(13) WFblue=CWUblueYield=10×ETblueYield

(14) $$ET_{blue}=\max(0,\;ET_{c}-P_{eff})$$

where *ET_c_*, *ET_green_*, and *ET_blue_* represent, respectively, total evapotranspiration, green water evapotranspiration, and blue water evapotranspiration (m^3^ ha^−1^) in citrus production annually. *ET_c_* was calculated by the CROPWAT model, which is listed in **Supplementary Figure S1**; *P_eff_* is the total effective rainfall used for citrus growth and was estimated and is shown in **Supplementary Figure S1**. The digital 10 is the conversion factor of water depth into water volume per unit area.

WF_grey_ represents water required diluting N and P pollution during citrus production and is mainly caused by the loss of fertilizers and pesticides. In this study, only loss of total N and P from fertilizer was accounted for, as the process of pesticides utility is difficult to detect. WF_grey_ can be calculated as the following:

(15) WFgrey=αN×ARNYield×(CN−max−CN−min)+αP×ARPYield×(CP−max−CP−min)

where αN and *α_P_* are the proportion of total N and P losses (by runoff and leaching) from chemical fertilizer. According to our previous research carried out in the same research region (Liu et al., 2021b), 46.22% N and 2.22% P were lost by runoff and leaching in clean tillage management, whereas 26.11% N and 1.37% P were lost in cropping management, respectively. AR is the chemical fertilizer application rate in kg ha^−1^. C_N-max_, C_N-min_ and C_P-max_, C_P-min_ (kg m^3^) refer to the maximum holding capacity of N and P in the water body and the concentration in the natural state, respectively. According to China’s environmental quality standard of surface level V water body (GB3838-2002), the highest permissible concentrations for total N (C_N-max_) and P (C_P-max_) were 2 and 0.4 mg L^−1^, and the minimum permissible concentrations for total N (C_N-min_) and P (C_P-min_) were 0 and 0 mg L^−1^, respectively.

Finally, the total WF was calculated by the sum of WF_green_, WF_blue_, and WF_grey_.

(16) $$WF=WF_{green}+WF_{blue}+WF_{grey}$$

### 2.6 Statistical analysis

Data processing was performed using Microsoft Office Excel 2016 and ArcGIS 10.0. The survey and experiment data were divided into four groups according to average CF (FCF or PCF) and citrus yield, including the low yield and low CF level (LL), low yield and high CF level (LH), high yield and low CF level (HL), and high yield and high CF level (HH) groups. All statistical analyses were performed using SPSS 17.0, and figures were performed using SigmaPlot 12.5. Significant differences of the FCF, PCF, carbon efficiency, and WF among different nutrient management strategies were analyzed using one-way ANOVA and least significant difference (LSD). The levels of significance were defined at *P* < 0.05 (*) and *P* < 0.01 (**). Spearman correlations between each WF and CF fractions were calculated using SigmaPlot 12.5.

## 3 Results

### 3.1 Inclusion of organic fertilizer and cover crop significantly increased farmer economic benefit

The resource inputs of different nutrient management strategies are shown in **Table 1**. The OPT group is a predefined integrated strategy aimed at utilizing localized fertilizer application amounts to fulfill the principles of the OSCF policy. Therefore, as its design objective indicates, the amount of chemical nitrogen and phosphorus fertilizers used for citrus production in the OPT group was less than that in the Che+CC group (*P* < 0.05) Due to the large variation in organic fertilizer inputs among farmers, there was no significant difference in organic nutrient inputs among the Che+Org, Che+Org+CC, and OPT groups (*P* < 0.05). The total N inputs of the Che+CC and Che+Org+CC groups were significantly greater than that of the OPT group (*P* < 0.05). In contrast, the OPT group consumed more fuel than the Che, Che+CC, and Che+Org+CC groups (*P* < 0.05). Although the chemical input was intentionally reduced, there was no significant difference in the citrus yield among the group. Moreover, the net economic benefit of farmers in the Che+CC, Che+Org+CC, and OPT groups were significantly greater than those in the Che and Che+Org groups (*P* < 0.05), demonstrating the feasibility of this design strategy.

### 3.2 Co-benefits of high yield and low CF with combined use of organic–inorganic fertilizers (OPT) with or without cover crop

**Figure 4:**
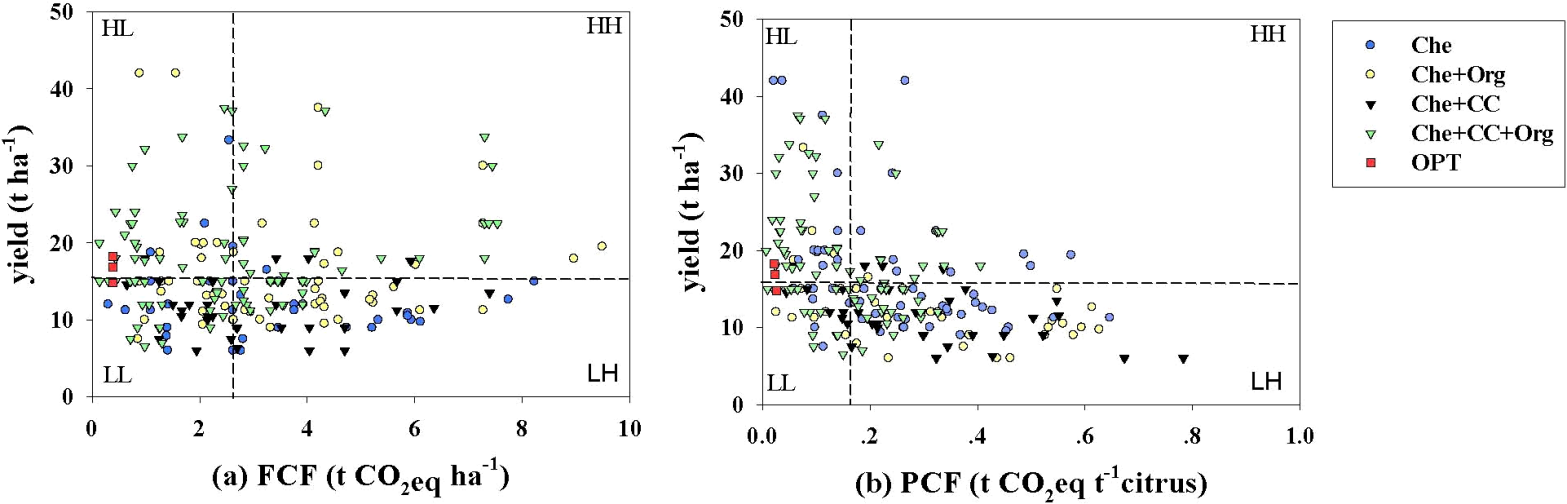
FCF and yield **(a)**, PCF and yield **(b)** scatter distributions. The farm CF (FCF) and product CF (PCF), expressed in terms of net GHG emissions per unit orchard area and per unit weight of fresh citrus yield. Che, Che+ Org, Che+ CC, Che+ Org+ CC, and OPT refer to chemical fertilizer only, chemical fertilizer plus organic fertilizer, chemical fertilizer plus cover crop, and chemical fertilizer plus combined organic material inputs and optimal nutrient management, respectively. The data on yield vs. carbon footprint were divided into four groups, namely, low yield and low emission (LL); low yield and high emission (LH); high yield and low emission (HL); and high yield and high emission (HH).

The survey data of 273 farms and 3 years of optimal management experiment data were divided into LL, LH, HL, and HH levels based on CF (FCF or PCF) and yield (**Figure 4**). The average FCFs in the LL, HL, LH, and HH levels were 0.51, 0.47, 4.12, and 4.81 t CO_2_ eq ha^−1^, respectively (**Figure 4a**). The average PCFs in the LL, HL, LH, and HH levels were 0.01, 0.004, 0.34, and 0.33 t CO_2_ eq t^−1^, respectively (**Figure 4b**). Approximately 47.5%, 42.1%, and 30.0% farms treated with Che, Che+Org, and Che+CC were all attributed to LH level (**Figure 4a**). However, approximately 51.2% farms treated with Che+CC+Org were attributed to HL level (**Figure 4a**). Two out of three OPT experiment fields were also in the HL level (**Figure 4a**). Although there are differences in data sources and sample sizes, this comparison remains valid because both sets of data used the same local emission factors and system boundaries and conducted completely identical LCA calculations. Thus, it is possible to fairly assess the performance gap between current practices and the optimized schemes based on scientific design.

### 3.3 Chemical N fertilizer followed by organic fertilizer contributed most of FCF and PCF

**Figure 5:**
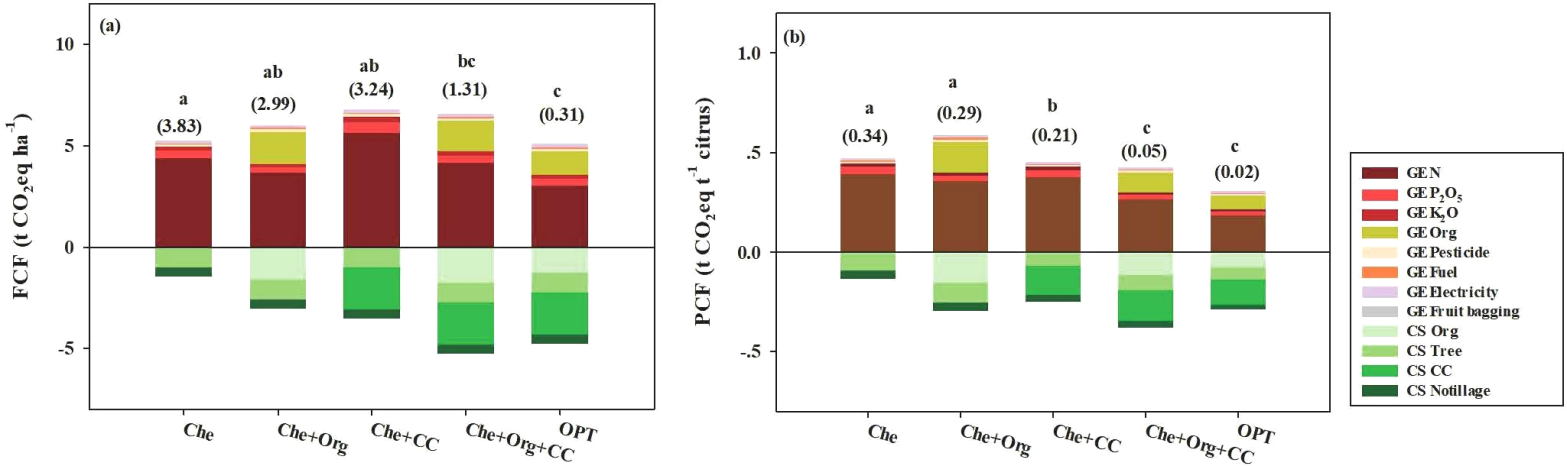
FCF, PCF, and the contribution of each input to FCF **(a)** and PCF **(b)** in orchards under different nutrient managements. The farm CF (FCF) and product CF (PCF), expressed in terms of net GHG emissions per unit orchard area and per unit of fresh citrus yield. Che, Che+ Org, Che+ CC, Che+ Org+ CC, and OPT refer to chemical fertilizer only, chemical fertilizer plus organic fertilizer, chemical fertilizer plus cover crop, and chemical fertilizer plus combined organic material inputs and optimal nutrient management, respectively. Different letters above each column indicate that there is a significant difference at 0.05 level, and numbers in brackets indicate net carbon footprint. GE N, GE P_2_O_5_, GE K_2_O, GE Organic fertilizer, GE Pesticide, GE Fuel, GE Electricity, and GE Fruit bagging refer to greenhouse gas emissions caused by nitrogen, phosphorus, potash, organic fertilizer, pesticides, fuel, electricity, and fruit bag production. CS Org, CS Tree, CS CC, and CS Notillage refer to the carbon sequestration by organic fertilizer, citrus tree, cover crop, and no tillage, respectively.

In the citrus field experiment of this study, the FCF ranged between 0.31 and 3.83 t CO_2_ eq ha^−1^, and the PCF between 0.02 and 0.34 t CO_2_ eq t^−1^ depending on the nutrient management treatment (**Figure 5**). The OPT showed significantly reduced FCF compared with the Che, Che+Org, and Che+CC groups (**Figure 5a**). This declining trend was more pronounced for PCF with OTP having the lowest value (**Figure 5b**). Also, the Che+CC and Che+Org+CC groups showed significant reductions in PCF compared with Che and Che+Org (**Figure 5b**).

Chemical nitrogen fertilizer use (GE_N_) accounted for 70.71% of greenhouse gas (GHG) emissions on average, making it the primary CF source in citrus production (**Figure 5**). The use of organic fertilizer (GE_Organic fertilizer_) and the use of phosphate fertilizer (GE_P2O5_) were the second and third CF contributors, accounting for 14.34% and 6.18% of GHG emissions, respectively (**Figure 4**). GEK_2_O, GE_Pesticides_, GE_Fuel_, GE_Electricity_, and GE_Fruit_ bagging accounted for 3.21%, 2.33%, 2.10%, 0.86%, and 0.28% of GHG emissions, respectively (**Figure 5a**). The main CF mitigation factors in the citrus system were soil organic carbon sequestration by cover crops (CSC), organic fertilizer use (CS_Org_), citrus tree pruning litter (CS_Litter_), and no-tillage management (CS_No-tillage_), and they accounted for 23.81%, 15.89%, 15.58%, and 7.21% of GHG emissions, respectively (**Figure 5a**). For GHG emissions per ton (citrus yield), nitrogen fertilizer, organic fertilizer, phosphate fertilizer, potassium fertilizer, electricity, pesticides, fuel oil, and paper bags contributed 70.22%, 14.58%, 6.10%, 3.18%, 2.48%, 2.24%, 0.91%, and 0.29%, respectively. Carbon sequestration by tree growth (CS_Tree_) and no-till practice offset 23.67%, 16.74%, 15.98%, and 7.80% (**Figure 5b**).

### 3.4 Different management and citrus varieties have greatly varied CF

There are significant differences between OPT management and farmers’ crop management practices, and it impacted both FCF and PCF (**Figure 5**). FCF was drastically diminished under OPT, with the reduction ranging from 76.34% to 91.91%, compared with the Che, Che+Org, Che+CC, and Che+Org+CC methods. In the OPT management, carbon emission was decreased by 3.18%, 15.23%, 24.86%, and 22.39% compared with Che, Che+Org, Che+CC, and Che+Org+CC, respectively, whereas OPT carbon sequestration was increased by 230.97% compared with Che. The PCF associated with cover crop management varied significantly between Che+CC, Che+Org+CC, and OPT compared with those without cover crop (Che, Che+Org). The PCF of OPT was 94.12%, 93.10%, 90.47%, and 60.00% lower than those of Che, Che+Org, Che+CC, and Che+Org+CC, respectively. Compared with PCF, the reduction in carbon emission of OPT was smaller, and it was 34.84%, 47.36%, 32.14%, and 27.67% lower than those of Che, Che+Org, Che+CC, and Che+Org+CC, respectively. However, carbon sequestration in the OPT group was increased by 121.71% and 18.21% compared with Che and Che+CC and reduced by 1.51% and 23.46% in Che+Org and Che+Org+CC, respectively.

**Figure 6:**
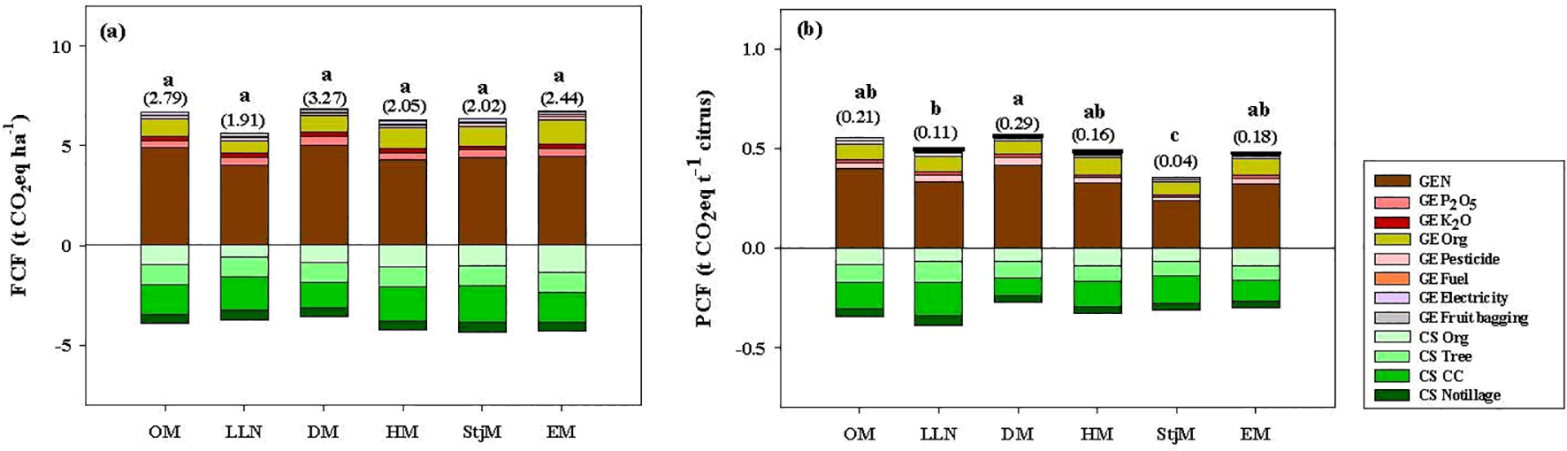
FCF, PCF, and the contribution of each input to FCF **(a)** and PCF **(b)** under different citrus varieties. The farm CF (FCF) and product CF (PCF), expressed in terms of net GHG emissions per unit orchard area and per unit of fresh citrus yield. OM, LLN, DM, HM, StjM, and EM refer to Orah mandarin, Lane Late Navel Orange, Daya mandarin, Harumi mandarin, Shatangju mandarin, and Ehime mandarin. Different letters after peer data indicate that the zone groups are significantly different at the 0.05 level, and numbers in brackets indicate net carbon footprint. GE N, GE P_2_O_5_, GE K_2_O, GE Organic fertilizer, GE Pesticide, GE Fuel, GE Electricity, and GE Fruit bagging refer to the greenhouse gas emissions caused by nitrogen, phosphorus, potash, organic fertilizer, pesticides, fuel, electricity, and fruit bag production, respectively. CS Org, CS Tree, CS CC, and CS Notillage refer to the carbon sequestration by organic fertilizer, tree, cover crop, and no tillage, respectively.

From the perspective for FCF, there is no significant difference between different citrus varieties that were cultivated under similar management methods (**Figure 6**). Compared with the FCF of varieties OM, LLN, DM, HM, and EM, FCF in StjM was decreased by 27.69%, −5.79%, 38.34%, 1.95%, and 16.60%, respectively. Also, StjM’s carbon emission, compared with OM, LLN, DM, HM, and EM, was decreased by 5.08%, −12.80%, 7.14%, −1.00%, and 5.79%, and carbon sequestration was increased by 11.17% and 16.60%, respectively. The PCF of StjM was significantly different from that of other varieties. (**Figure 6**). Compared with OM, LLN, DM, HM, and EM, the PCF of StjM was reduced by 80.00%, 63.64%, 85.71%, 75.00%, and 77.77%, respectively. Carbon emission of StjM was 35.48%, 28.33%, 36.66%, 26.54%, and 25.48% lower than those of OM, LLN, DM, HM, and EM, respectively. In contrast, StjM’s carbon sequestration was increased by 9.48% and 19.73% compared with OM, LLN, DM, HM, and EM.

**Figure 7:**
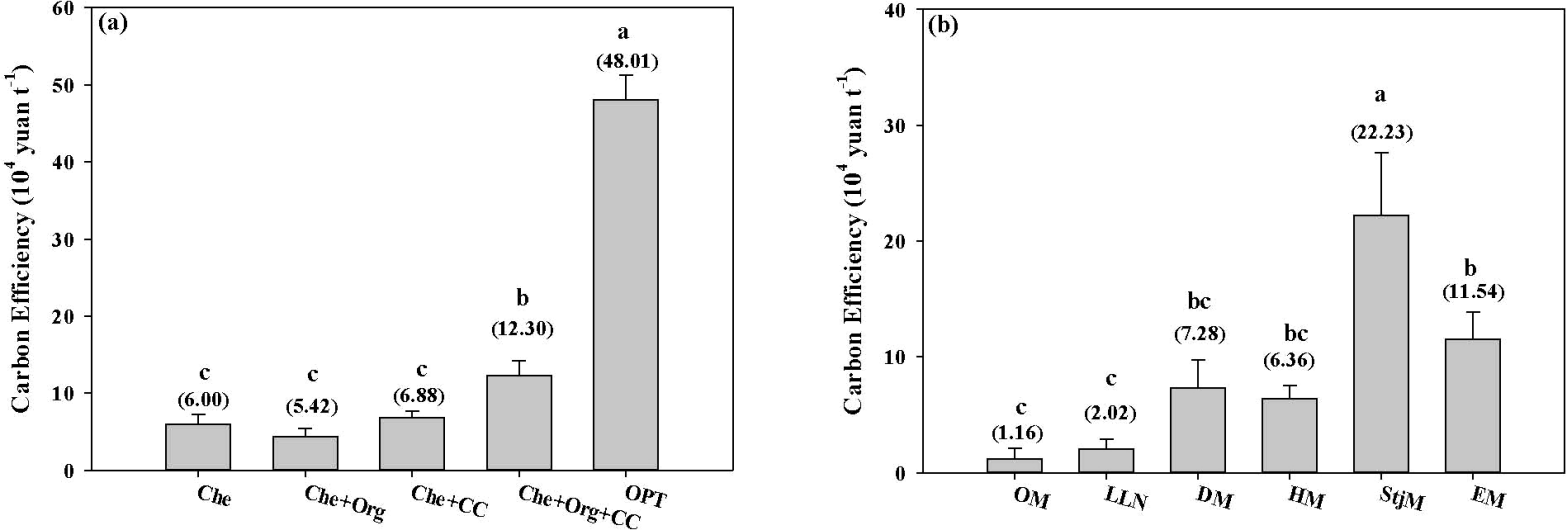
Carbon efficiency under different nutrient management **(a)** and citrus varieties **(b)**. Che, Che+ Org, Che+ CC, Che+ Org+ CC, and OPT refer to chemical fertilizer only, chemical fertilizer plus organic fertilizer, chemical fertilizer plus cover crop, and chemical fertilizer plus combined organic material inputs and optimal nutrient management, respectively. OM, LLN, DM, HM, StjM, and EM refer to Orah mandarin, Lane Late Navel Orange, Daya mandarin, Harumi mandarin, Shatangju mandarin, and Ehime mandarin, respectively. Different letters after peer data indicate that the zone groups are significantly different at the 0.05 level, and numbers in brackets indicate net carbon efficiency.

Orchards managed by OPT and Che+Org+CC achieved significantly higher carbon efficiency compared with other management methods (**Figure 7a**). The carbon efficiency of orchards managed by OPT was 8.00-, 8.86-, 6.98-, and 4.00-fold greater than those of Che, Che+Org, Che+CC, and Che+Org+CC, respectively. Among the varieties studied, StjM had the highest carbon efficiency (22.23 × 104 Yuan t^−1^ CO_2_eq) and it was 1.93, 3.05, 3.50, 11.00, and 19.16 times higher than those of EM, DM, HM, LLN, and OM, respectively (**Figure 7b**).

### 3.5 Co-benefits of significantly reduced GHG emissions and greater carbon sequestration under OPT and Che+Org+CC management

**Figure 8:**
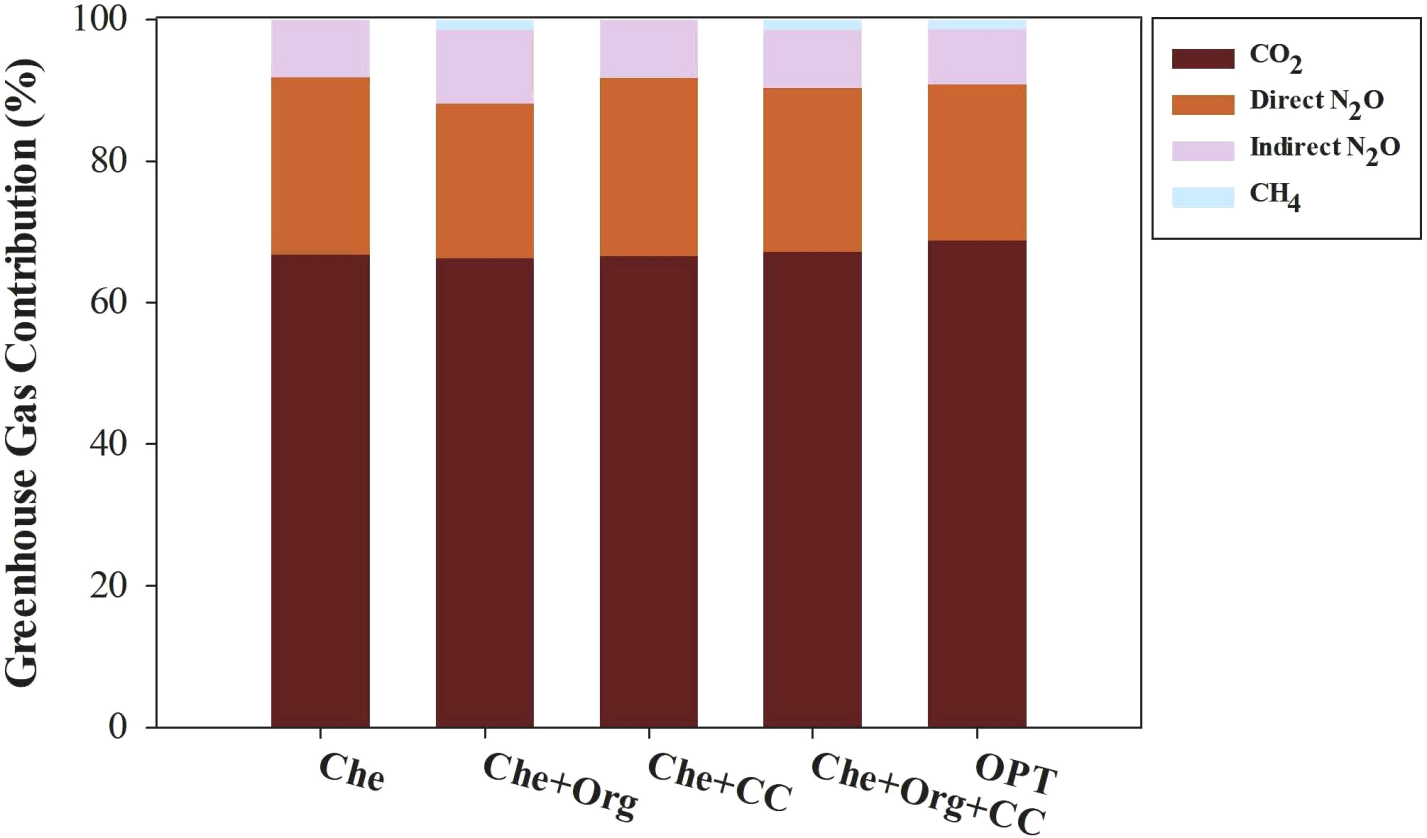
Composition of greenhouse gas emissions from citrus orchards under different nutrient managements. Che, Che+ Org, Che+ CC, Che+ Org+ CC, and OPT refer to chemical fertilizer only, chemical fertilizer plus organic fertilizer, chemical fertilizer plus cover crop, and chemical fertilizer plus combined organic material inputs and optimal nutrient management, respectively.

Greenhouse gas emissions from agricultural operations in citrus cultivation include CO_2_ emissions from agricultural material production, direct N_2_O emissions from N_2_O escape caused by nitrogen fertilizer use, indirect N_2_O produced from NH_3_ emission and NH_4_ leaching, and methane emissions from organic fertilizer application. In our experiments, CO_2_ caused the most significant greenhouse effect, followed by the direct and indirect emissions of N_2_O and CH_4_ (**Figure 8**). Che, Che+Org, Che+CC, Che+Org+CC, and OPT account for 66.89%, 66.27%, 66.66%, 67.21%, and 67.21%, respectively, of the greenhouse effect caused by CO_2_ emissions. Direct N_2_O was estimated to contribute 24.97%, 21.86%, 25.14%, 23.17%, and 22.03% greenhouse effect in Che, Che+Org, Che+CC, Che+Org+CC, and OPT, respectively. The greenhouse effect caused by indirect N_2_O was 8.14%, 10.44%, 8.20%, 8.20%, and 7.83% for Che, Che+Org, Che+CC, Che+Org+CC, and OPT, respectively. Methane emitted by Che+Org, Che+Org+CC, and OPT account for 1.43%, 1.42%, and 1.32% of greenhouse effect in Che+Org, Che+Org+CC, and OPT, respectively.

**Figure 9:**
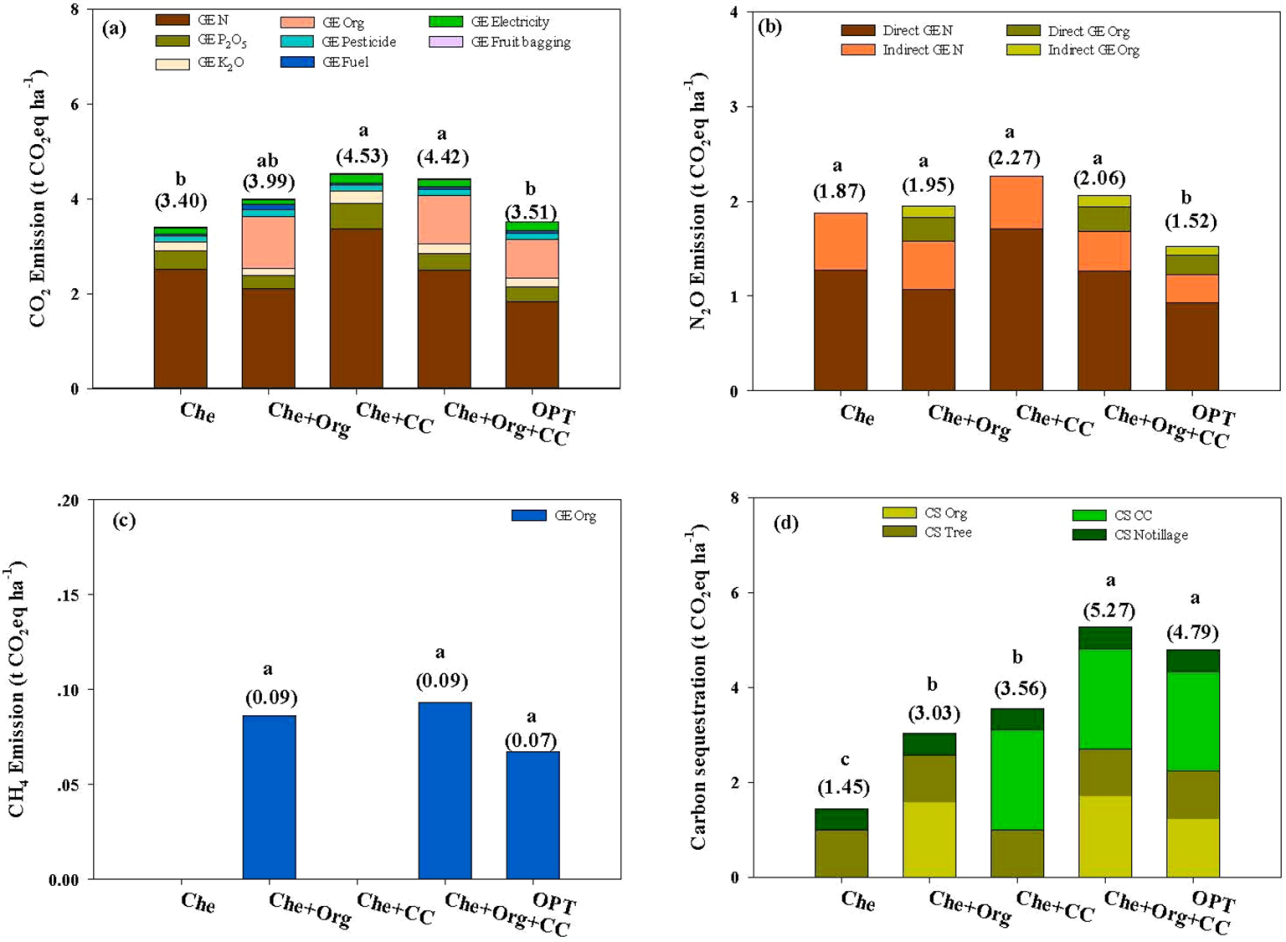
Effects of different nutrient management on CO_2_ emissions **(a)**, N_2_O emissions **(b)**, CH_4_ emissions **(c)**, and carbon sequestration **(d)** per unit area of citrus orchards. Che, Che+ Org, Che+ CC, Che+ Org+ CC, and OPT refer to chemical fertilizer only, chemical fertilizer plus organic fertilizer, chemical fertilizer plus cover crop, and chemical fertilizer plus combined organic material inputs and optimal nutrient management, respectively. Different letters after peer data indicate that the zone groups are significantly different at the 0.05 level, and numbers in brackets indicate net carbon emission or net carbon sequestration. GE N, GE P_2_O_5_, GE K_2_O, GE Organic fertilizer, GE Pesticide, GE Fuel, GE Electricity, and GE Fruit bagging refer to the greenhouse gas emissions caused by nitrogen, phosphorus, potash, organic fertilizer, pesticides, fuel, electricity, and fruit bag production, respectively. Direct GE N, Indirect GE N, Direct GE Org, and Indirect GE Org refer to direct & indirect GHG emissions from nitrogen fertilizers and direct & indirect GHG emissions from organic fertilizers, respectively. CS Org, CS Tree, CS CC, and CS Notillage refer to the carbon sequestration by organic fertilizer, tree, cover crop, and no tillage, respectively.

**Figure 10:**
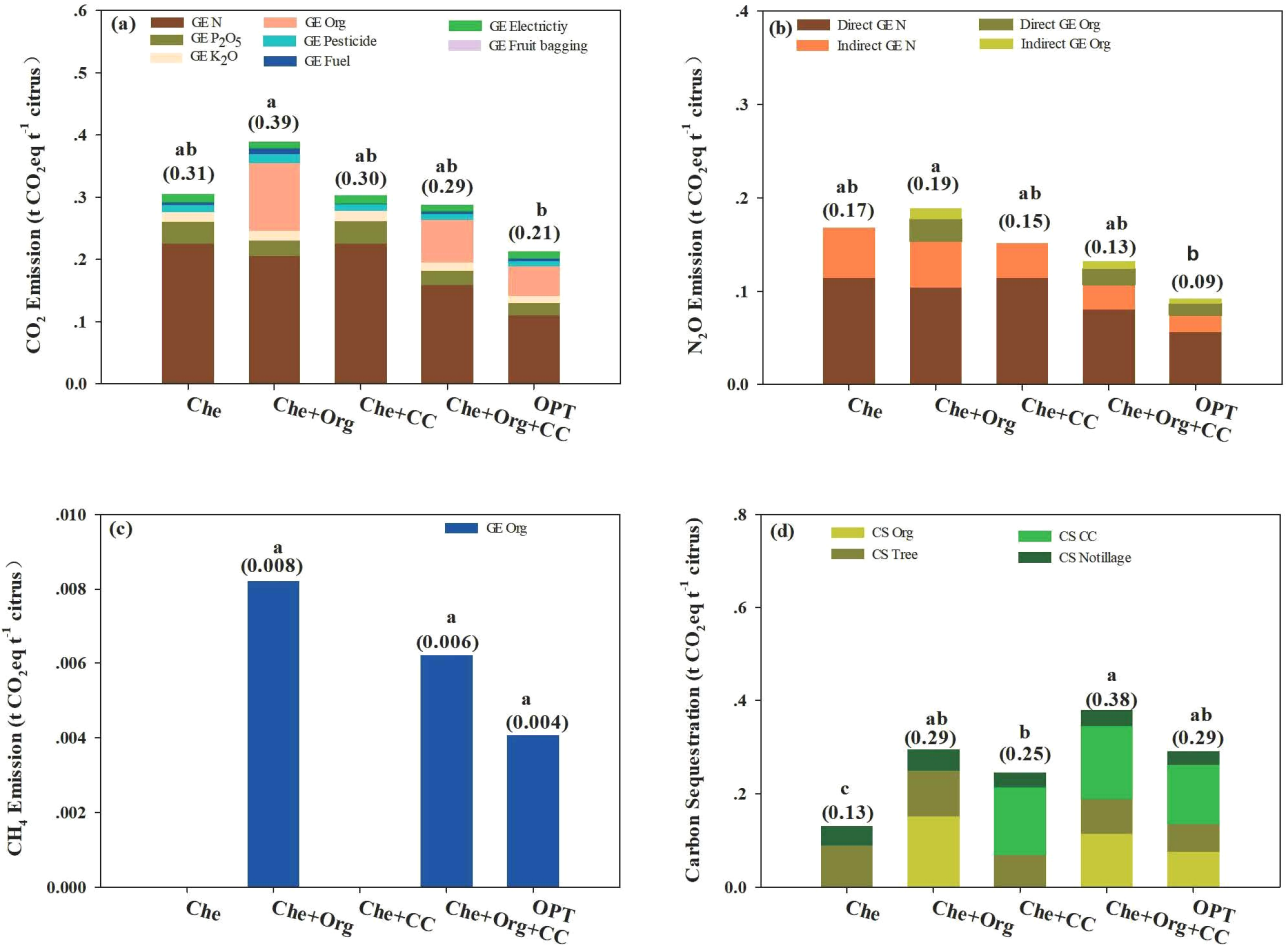
Effects of different nutrient management on CO_2_ emissions **(a)**, N_2_O emissions **(b)**, CH_4_ emissions **(c)**, and carbon sequestration **(d)** per unit yield of citrus orchards. Che, Che+ Org, Che+ CC, Che+ Org+ CC, and OPT refer to chemical fertilizer only, chemical fertilizer plus organic fertilizer, chemical fertilizer plus cover crop, and chemical fertilizer plus combined organic material inputs and optimal nutrient management. Different letters after peer data indicate that the zone groups are significantly different at the 0.05 level, and numbers in brackets indicate net carbon emission or net carbon sequestration. GE N, GE P_2_O_5_, GE K_2_O, GE Organic fertilizer, GE Pesticide, GE Fuel, GE Electricity, and GE Fruit bagging refers to the greenhouse gas emissions caused by nitrogen, phosphorus, potash, organic fertilizer, pesticides, fuel, electricity, and fruit bag production. Direct GE N, Indirect GE N, Direct GE Org, and Indirect GE Org refer to direct and indirect GHG emissions from nitrogen fertilizers and direct and indirect GHG emissions from organic fertilizers. CS Org, CS Tree, CS CC, and CS Notillage refer to the carbon sequestration by organic fertilizer, tree, cover crop, and no tillage.

Orchards under OPT management reduced CO_2_ and N_2_O emissions and increased carbon sequestration, resulting in much lower FCF and PCF compared with other management methods evaluated in this study (**Figures 9**, **10**). Fertilizer is still the primary source of greenhouse gas emissions in FCF. The greenhouse effect of CO_2_ emitted from fertilizer production and application ranged from 3.08 to 4.16 t CO_2_eq ha^−1^, accounting for 89.06% to 92.11%. Additionally, N_2_O and methane emissions are also associated with fertilizer application (**Figure 9**). Similarly, fertilizer remains the primary source of greenhouse gas emissions in PCF. The greenhouse effect of CO_2_ emitted from fertilizer production and application ranged from 0.19 to 0.35 t CO_2_eq ha^−1^. N_2_O and methane emissions were also attributed to fertilizer application (**Figure 10**).

### 3.6 The WF and its relationship with CFs

As shown in **Figure 3**, there was no significant difference for blue and green water footprints among the management models tested here. Compared with Che and Che+Org, OPT’s blue water footprint was reduced by 4.63% and 13.10%, respectively. The green water footprint of OPT was reduced by 29.39% and 35.66%, respectively, when compared with Che and Che+Org.

The grey water footprint crop cover management models (Che+CC, Che+Org+CC, and OPT) is significantly different from those without cover crop (Che and Che+Org). Crops in OPT had their grey water footprint decreased by 71.58%, 67.98%, 50.33%, and 28.60%, respectively, compared with Che, Che+Org, Che+CC, and Che+Org+CC. The overall water footprint of OPT was reduced by 67.98%, 64.62%, 45.97%, and 26.94%, respectively, when compared with Che, Che+Org, Che+CC, and Che+Org+CC.

**Figure 11:**
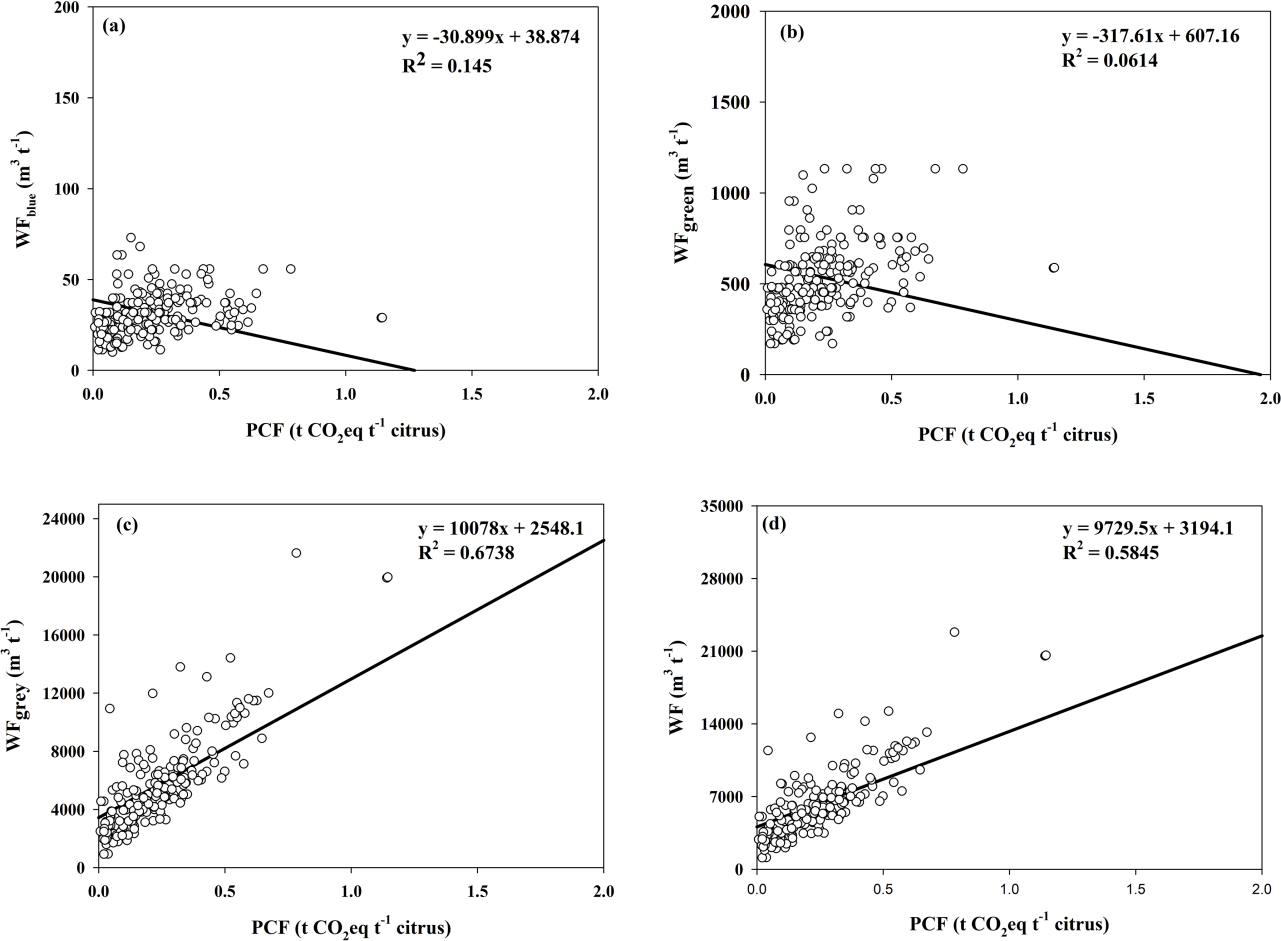
Correlation analysis of PCF and blue water footprint **(a)**, PCF and green water footprint **(b)**, PCF and grey water footprint **(c)**, and PCF and total water footprint **(d)** of citrus orchards. The product CF (PCF), expressed in terms of net GHG emissions per unit of fresh citrus yield. WFblue represents the fresh surface or groundwater that either evaporates, is incorporated into the product, and does not return to the same catchment area. WFgreen refers to the precipitation that is stored in or stays on top of the soil. WFgrey is defined as the volume of freshwater that is required to assimilate the load of pollutants based on natural background concentrations and existing ambient water quality standards.WF means the total water footprint.

As shown in **Figure 11**, PCF is negatively correlated with the blue and green water footprints; that is, the higher the PCF, the smaller the blue and green water footprints. An opposite trend was evident for PCF and the grey water footprint and overall water footprint.

## 4 Discussion

### 4.1 CF contribution factors

In 2020, China’s agricultural carbon emissions amounted to 856 million tons of CO_2_eq (FAO, 2022). Among them, the fruit industry, especially citrus production, has become a significant contributor to GHG emissions due to its extensive planting area and high resource input. Its carbon emission intensity is notably higher than the global average (Lin et al., 2015; Yan et al., 2016).

Fertilizers, especially nitrogen fertilizers, are the primary source of CF in agriculture. This study is consistent with the conclusions of most studies, confirming that the production and application of chemical fertilizers are the main contributors to CF, with a contribution rate ranging from 68.16% to 94.49%. Among them, the emission of nitrogen fertilizer accounts for as high as 88.28% (Chen et al., 2020; Yan et al., 2016). Previous studies have also identified a positive correlation between nitrogen fertilizer input and carbon emissions (Chen et al., 2020). Nitrogen use efficiency in Chinese agriculture is relatively low, with nitrogen fertilizer application rates practiced by farmers, including for citrus, being excessively high. Additionally, nitrogen fertilizer partial productivity is only approximately 40% of that of developed countries (Chen et al., 2020; Yang et al., 2020). Therefore, the primary focus for reducing carbon emissions in orchards should be on decreasing nitrogen use and enhancing nitrogen use efficiency. From our study, it is evident that even if the amount of nitrogen applied is greatly reduced, the fruit yield can be maintained at a high level. In general, a reduction of nitrogen input by 30.0% can result in a reduction of greenhouse gas emissions by 15.0% to 24.0% (Yan et al., 2016). The OPT model in this study has been verified in practice to achieve significant emission reduction while maintaining high yields through optimized management.

The use of organic fertilizers significantly reduced the farm carbon footprint (FCF) and product carbon footprint (PCF), which is consistent with the findings of Ribal et al. (2016) and Aguilera et al. (2014). However, our FCF and PCF values were notably higher than those reported by Martin-Gorriz et al. (2020). Through the research conducted by Martin-Gorriz et al. (2020), it provides a suitable reference for analyzing the mechanism of the impact of fertilization management on carbon footprint. Because this discrepancy primarily stems from differences in nitrogen input level, the lemon, citrus, and orange trees in Martin-Gorriz et al. (2020) received substantially lower nitrogen fertilization rates than those used in our study. Our results further demonstrate that chemical fertilizers constitute the primary source of agricultural carbon emissions, with a positive correlation observed between nitrogen application rates and carbon emissions. Consequently, the variance in fertilization rates became the key determinant explaining the higher PCF and FCF values in our study relative to that of Martin-Gorriz et al. (2020).

Apart from chemical fertilizers, organic fertilizers are a significant source of GHG in orchards. However, organic fertilizers can also increase soil organic carbon in orchards through carbon sequestration, which helps alleviate GHG. In this study, the carbon sink benefit of organic fertilizer outweighed its carbon emission attribute. The addition of 10 t ha^−1^ (dry weight) of organic fertilizer can significantly enhance the carbon sequestration rate (Aguilera et al., 2013). Zhou et al. (2022) reported that increasing the application ratio of organic fertilizer to chemical fertilizer enhances the utilization efficiency of the latter, leading to improved yield and quality of citrus.

Previous studies on CFs have primarily focused on GHG emissions caused by agricultural production activities. These include direct GHG emissions from the production of agricultural products such as fertilizers (Chen et al., 2015; Li et al., 2016; Clark et al., 2016; Pishgar-Komleh et al., 2013), direct greenhouse gas emissions from fertilizer application (Gu et al., 2019; Zhang et al., 2017), and indirect greenhouse gas emissions from soil denitrification (Zhang et al., 2017; Liu et al., 2021a; Kang et al., 2022). However, there are limited studies on carbon sequestration, which includes the CO_2_ absorbed through photosynthesis during crop growth and development (Wu, 2008), as well as the increase in SOC resulting from practices like cover crops, organic fertilizers (Aguilera et al., 2013; Yang et al., 2020), and no tillage to reduce SOC decomposition (Lu et al., 2009). Cover crops are an important component of soil carbon sequestration and the most significant component that mitigates CF. Carbon input from cover crops is transferred from the atmosphere to the soil through photosynthesis, increasing SOC (Blanco-Canqui, 2017). Additionally, they reduce runoff losses and soil erosion in steep-slope orchards significantly (Ledo et al., 2020; Liu et al., 2021a). This practice can decrease the amount of both soil carbon and nitrogen leaving the orchard system through runoff, ultimately reducing orchard CF (Repullo-Ruibérriz de Torres et al., 2018).

### 4.2 The interaction between WF and CF

The study area is a typical hilly landform experiencing serious soil erosion and low fertilizer utilization. Among the issues, a low nitrogen utilization efficiency leads to a high carbon footprint and a high grey water footprint. The greenhouse effect, as well as the eutrophication of rivers and lakes, is largely caused by the excessive use of nitrogen fertilizers (Liu et al., 2013). The loss of chemical nitrogen and phosphate fertilizers causes grey water footprint, and thus reducing the grey water footprint indirectly promotes the utilization of fertilizers. Moreover, because the research results (**Figure 11**) show that the carbon footprint (CF) is significantly positively correlated with the grey water footprint and the overall water footprint, reducing the grey water footprint indirectly reduces the carbon footprint.

Secondly, cover crops are a significant contributor to the reduction in the grey water footprint, as they substantially reduce the loss of nitrogen and phosphorus, as much as 53.4% and 56.9%, respectively, as reported in a previous study (Liu et al., 2021a). The grey water footprint varies significantly based on management practices, and it was significantly lower in cover-cropped management models (Che+CC, Che+Org+CC, and OPT) than those without cover crop (Che and Che+Org). Additionally, OPT-managed orchards have a significantly lower grey water footprint compared with the models using farmer practice. The overall water footprint (WF) under OPT management ranged between 32.02% and 73.06%. In addition, cover crops increased the green water footprint, but the increase was smaller than the decrease in grey water footprint. The overall water footprint of OPT was significantly lower than that of other models tested.

In different nutrient management models studied here, both CFs and the overall water footprint were influenced by environmental factors and farming practices. As shown in **Figure 3**, there was little difference in the blue water and green water footprints across different management models. Blue water and green water are mainly affected by local climatic conditions and crop varieties. The CF is significantly positively correlated with the grey water footprint and the overall water footprint. The grey water foot was however the most important factor affecting the CFs of citrus production. By reducing application of chemical fertilizers and cover crop management between tree rows, grey water footprint can be greatly reduced, thereby reducing the CFs of citrus production.

### 4.3 Effects of different management models on CFs

Organic fertilization and cover crop management practices have emerged as promising strategies to reduce GHG emissions and mitigate global warming. In this context, we studied five distinct nutrient management methods (Che, Che+Org, Che+CC, Che+Org+CC, and OPT) and found that the OPT method performed the best in terms of environmental and economic benefits. The fundamental reason for this is that OPT does not simply add organic inputs or cover crops; instead, it achieves a synergistic enhancement of nutrient cycling and carbon sequestration functions through systematic optimization. (**Figure 5**).

The results of our study indicate that OPT can reduce FCF to 0.31 t CO_2_ eq ha^−1^ and PCF to 0.02 t CO_2_ eq ha^−1^. In comparison with other studies, our study demonstrates relatively lower CFs of citrus production though with notable disparities (**Table 4**). These differences can be attributed to several factors: Firstly, our approach involved not only reducing the application of chemical fertilizers but also increasing investment in organic fertilizers and implementing cover crop management. Secondly, while other studies typically employ IPCC-recommended values for carbon emission parameters, our study optimized these parameters specifically for citrus production, incorporating information obtained from local experiments. This refinement enhances the accuracy of carbon emissions estimates for Chinese citrus production. For example, the estimated NH_3_ volatilization (EFChe-NH_3_) and N leaching (EFChe-RL) in dryland apple orchards in northern China were 10.8% (Ge et al., 2011) and 10.0% of total chemical N fertilizer inputs, whereas the emission parameter EFChe-N2O obtained by field tests in the study area is 13.38% (Kang et al., 2022). EFChe-RL was 46.22% without cover crops and 26.11% with cover crops (Liu et al., 2021a). Moreover, while our study fully considered the role of carbon sequestration, some studies (Ribal et al., 2016; Maestre-Valero et al., 2018; Mazis et al., 2021) neglect to account for greenhouse gas offset by carbon sequestration, resulting in higher CF values than what was observed in our study. Additionally, other studies’ boundary systems (Maestre-Valero et al., 2018) encompass CF resulting from irrigation, a factor not included in our study as there was no irrigation in our production system, thereby contributing to difference in CFs. The citrus boundary system studied by Yan et al. (2016) includes CF influenced by irrigation, leading to a slightly larger CF than ours. Furthermore, variations in fertilizer quantities, as observed in other studies (Chen et al., 2020; Maestre-Valero et al., 2018; Ribal et al., 2019), lead to differing CF levels. Additionally, the citrus boundary system examined by Li et al. (2021) encompasses five links, four more than in our study: post-harvest processing, storage, transportation, and consumption, resulting in significantly higher CF values than our findings. Lastly, the difference in climate can also influence CF levels (Aguilera et al., 2014). For instance, in a study in the Mediterranean climate region (Martin-Gorriz et al., 2020), the emphasis on reducing CO_2_ emissions resulted in the system functioning as an ideal carbon sink, with protective measures implemented over 7 years leading to an optimal CF state.

**Table 4:** Carbon footprint across global citrus production reported in recent literature.

Nation	Province	Citrus species	Management	Yield (t ha^-1^)	FCF (t CO_2_ eq ha^-1^)	PCF (t CO_2_ eq t^-1^)	Reference
Spain	Valencia	Citrus	Conventional	33.35	5.57	0.31	Ribal J., et al. (2016)
Spain	Valencia	Citrus	Organic	18.32	1.17	0.10	Ribal J., et al. (2016)
Spain	CampotéjarMurcia	Pomelo	–	76.86	5.68	0.07	Maestre-Valero J F, et al. (2018)
Spain	–	Citrus	Conventional	41.95	2.44	0.06	Aguilera E., et al. (2014).
Spain	–	Citrus	Organic	21.26	0.63	0.03	Aguilera E., et al. (2014).
Spain	Murcia	Lemon	–	29.50	-18.88	-0.64	Martin-Gorriz B., et al. (2020)
Spain	Murcia	Citrus	–	22.50	0.68	-0.03	Martin-Gorriz B., et al. (2020)
Spain	Murcia	Orange	–	20.89	-2.92	-0.14	Martin-Gorriz B., et al. (2020)
Spain	–	Orange	Conventional	34.25	28.09	0.82	Ribal et al. (2019)
Spain	–	Orange	Organic	10.49	7.03	0.67	Ribal et al. (2019)
Australia	–	Orange	–	–	–	0.22	Bell E and Horvath A (2020)
Mexico	–	Orange	–	–	–	0.33	Bell E and Horvath A (2020)
Australia	–	Orange	–	–	–	0.22	Bell E and Horvath A (2020)
South Africa	–	Orange	–	–	–	0.36	Bell E and Horvath A (2020)
Chile	–	Orange	–	–	–	0.40	Bell E and Horvath A (2020)
Greece	Arta Prefecture	Orange	–	53.65	6.98	0.13	Mazis A, et al. (2021)
Lebanon	–	orange	–	21.48	17.20	0.80	Skaf L, et al. (2019)
Lebanon	–	Citrus	–	25.85	28.00	1.08	Skaf L, et al. (2019)
American	Texas	Orange	–	–	–	0.22	Bell E and Horvath A (2020)
American	Florida	Orange	–	–	–	0.26	Bell E and Horvath A (2020)
China	Sichuan	Citrus	–	24.40	11.67	0.64	Yang M, et al. (2020)
China	Fujian	Pomelo	–	56.30	16.50	0.33	Chen X, et al. (2020)
China	Hubei	Orange	–	56.00	7.10	0.14	Yan M, et al. (2016)
China	Guangxi	Citrus	–	42.58	100.07	2.91	Li et al., (2021)
China	Chongqing	Citrus	Che	12.45	3.83	0.34	This study
China	Chongqing	Citrus	Che+ Org	11.13	2.99	0.29	This study
China	Chongqing	Citrus	Che+ CC	16.94	3.24	0.21	This study
China	Chongqing	Citrus	Che+ Org+ CC	17.76	1.31	0.05	This study
China	Chongqing	Citrus	OPT	16.63	0.31	0.02	This study

The farm CF (FCF) and product CF (PCF), expressed in terms of net GHG emissions per unit orchard area and per unit of fresh citrus yield. Che, Che+ Org, Che+ CC, Che+ Org+ CC and OPT refer to chemical fertilizer only, chemical fertilizer plus organic fertilizer, chemical fertilizer plus cover crop, and chemical fertilizer plus combined organic material inputs and optimal nutrient management, respectively.

Among the 273 smallholder or large-scale citrus agribusinesses examined in this study (Che, Che+CC, Che+Org, and Che+Org+CC), both FCF and PCF were markedly higher than those observed in the 2-year OPT trial. This difference primarily arises from the intensive management practices involving high fertilizer and pesticide inputs prevalent in the study region. This stands in stark contrast to the findings of Zhang et al. (2022) on grain crop production in plain areas of China, where large-scale agriculture reduces the trade-offs between crop productivity and resource input, enhancing energy efficiency and achieving the co-benefits of high crop productivity and low energy consumption. The citrus cash crops in our study area predominantly grow in relatively steep hilly and mountainous terrains, facing challenges such as limited mechanization and higher labor cost. Farmers or large landowners typically rely on high resource inputs to attain high yields and economic returns. Knudsen’s (2011) and Beccali’s (2009) studies further corroborate that GHG emissions from citrus orchards used for fruit juice production with low intensification are lower compared with those from orchards employing intensive agricultural practices to increase fruit yield. Hence, to foster green and low-carbon development of citrus production in our study area, it is imperative to adopt low-intensity OPT management practices.

In our study, there was no significant difference in FCF among different citrus varieties, yet substantial disparities in PCF were evident. StjM and LLN varieties exhibited lower PCF compared with other varieties, primarily due to their higher yields. Therefore, cultivating varieties with relatively high yields such as StjM and LLN in this region while phasing out varieties with high carbon emissions proves to be an effective strategy for promoting the green development of regional citrus.

### 4.4 Limitations of the study

Although our research provides strong evidence for the benefits of integrated management in mountain citrus orchards, some limitations must also be acknowledged. Firstly, the CF and WF evaluations rely on the combination of farmer survey data and controlled experimental data from OPT treatments. Although we applied unified emission factors and system boundaries, the differences in management precision, tree age, and micro-topography among the surveyed farms, when directly compared with the optimization experiments, may introduce some uncertainties. Secondly, our carbon sink calculation uses conversion coefficients based on meta-analysis of organic inputs and cover crops. Although these values are derived from regional or global studies, long-term measurements of SOC dynamics specific to the location will further refine the accuracy of carbon sink quantification. Thirdly, this study focuses on the farm gate. Incorporating downstream processes (such as transportation and processing) into the full life cycle assessment can provide a more comprehensive view of the environmental impact of citrus production, although this exceeds the scope of this farm management comparison. Finally, the economic analysis considers direct costs and benefits. A more detailed cost–benefit analysis, covering labor input for cover crop management and potential long-term soil health benefits, will strengthen its practicality assessment.

## 5 Conclusion

In conclusion, the main source of CF in citrus orchards is the production and use of nitrogen fertilizer. Direct and indirect losses of nitrogen fertilizer, including losses during production and transportation processes, as well as nitrogen leaching, are the primary reasons for greenhouse gas emissions in citrus cultivation, increasing the CF. Using the life cycle assessment (LCA) method, the CF of five different crop management models, namely, Che, Che+Org, Che+CC, Che+Org+CC, and OPT, were calculated and compared. It was found that the OPT management model showed significant differences compared with other models. This is attributed to the use of partial chemical fertilizer substitution by organic fertilizer and cover crops in the OPT model. Therefore, the study concludes that organic fertilizer substitution and cover crops have a significant role in reducing the CF of citrus orchards, with the CF of the model using cover crop management significantly lower than other management models. Cover crops also play a key role in reducing the grey WF by significantly reducing nitrogen and phosphorus losses. The grey WF of crop management with cover crop was significantly lower than those without. Since the CF is significantly positively correlated with the grey WF and the total WF, cover crop management not only reduces the grey WF and the total WF but also lowers the CF. Deeper understanding of the key role of organic fertilizer substitution and cover crops in regulating CF and WF will further help reduce greenhouse gas emissions, thus improving the potential environmental impact of citrus production.

## Data Availability

The datasets presented in this study can be found in online repositories. The names of the repository/repositories and accession number(s) can be found in the article/**Supplementary Material**.
