# Correction: Co-benefits of reduced carbon and water footprints and enhanced carbon sequestration with integrated organic–inorganic fertilization and cover cropping in hilly citrus orchards

**DOI:** 10.3389/fpls.2026.1904009

**Published:** 2026-07-30

**Authors:** Wenwen Ning, Jian Zhao, Prakash Lakshmanan, Tieguang He, Shuai Wang, Yuanlong Ran, Pengjie Zhan, Zeyu Wang, Sili Ye, Yu Xiang, Yi Wen, Xiaojun Shi, Jingkun Zhao, Yuting Zhang

**Affiliations:** 1 College of Resources and Environment, Interdisciplinary Research Center for Agriculture Green Development in Yangtze River Basin, Southwest University, Chongqing, China; 2 College of Elementary Education, Chongqing Normal University, Chongqing, China; 3 Sugarcane Research Institute, Guangxi Academy of Agricultural Sciences, Nanning, China; 4 Agricultural Resources and Environmental Research Institute, Guangxi Academy of Agricultural Sciences/Guangxi Key Laboratory of Arable Land Conservation, Nanning, China; 5 Chongqing Agricultural Technology Extension Station, Chongqing, China; 6 Zhongxian Agricultural Science and Technology Extension Center, Chongqing, China

**Keywords:** carbon footprint, hilly citrus orchard, life cycle assessment, optimal nutrient management, water footprint

Author Prakash Lakshmanan was erroneously assigned to affiliation “Queensland Alliance for Agriculture and Food Innovation, The University of Queensland, St Lucia, QLD, Australia”. This affiliation has now been removed.

For author Tieguang He, was erroneously assigned the affiliation “Sugarcane Research Institute, Guangxi Academy of Agricultural Sciences, Nanning, China”. The correct affiliation is “Agricultural Resources and Environmental Research Institute, Guangxi Academy of Agricultural Sciences/Guangxi Key Laboratory of Arable Land Conservation, Nanning, China”.

The order of authors in the author list of the published paper was erroneously given as:

“Wenwen Ning, Jian Zhao, Prakash Lakshmanan, Shuai Wang, Yuanlong Ran, Tieguang He, Pengjie Zhan, Zeyu Wang, Sili Ye, Yu Xiang, Yi Wen, Xiaojun Shi, Jingkun Zhao and Yuting Zhang”.

The correct author list is:

“Wenwen Ning, Jian Zhao, Prakash Lakshmanan, Tieguang He, Shuai Wang, Yuanlong Ran, Pengjie Zhan, Zeyu Wang, Sili Ye, Yu Xiang, Yi Wen, Xiaojun Shi, Jingkun Zhao and Yuting Zhang”.

The original version of this article has been updated.

