# Supplementary material for: Co-benefits of reduced carbon and water footprints and enhanced carbon sequestration with integrated organic–inorganic fertilization and cover cropping in hilly citrus orchards
Source: Front Plant Sci. 2026 Feb 25;17:1763629. doi: 10.3389/fpls.2026.1763629 (PMC12975874; doi:10.3389/fpls.2026.1763629)
Supplement: Supplementary file 1 [file DataSheet1.pdf]

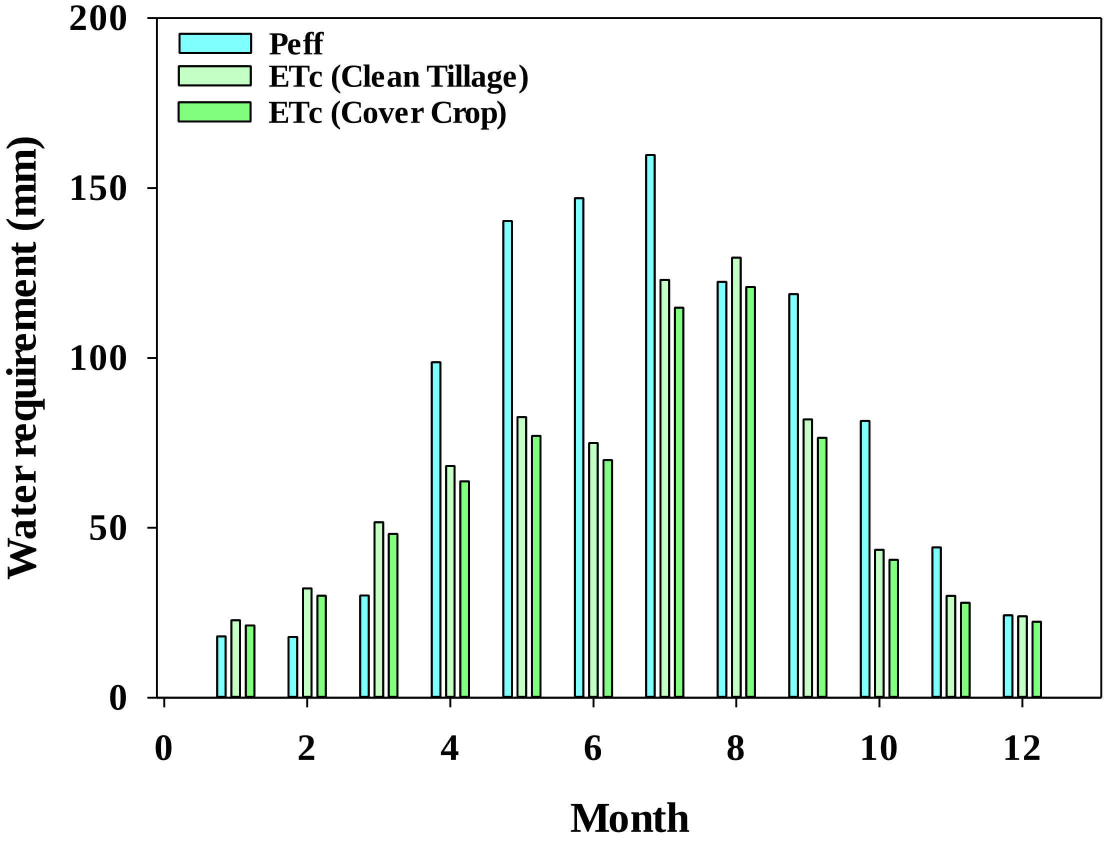

Fig.S1 Monthly distribution of Peff and ETc.

Note: Peff is the total effective rainfall for citrus growth, ETc is the evapotranspiration in citrus orchard.

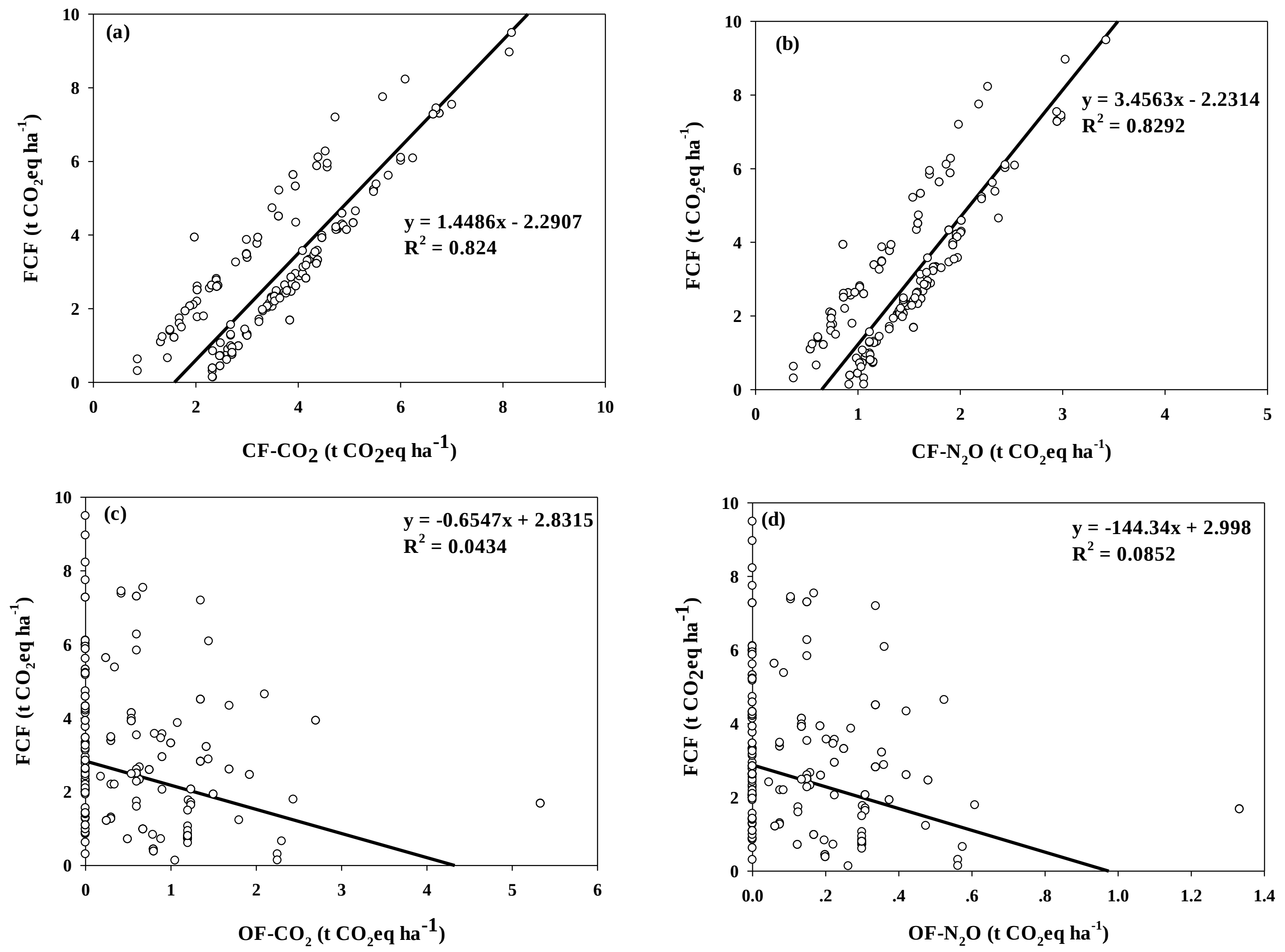

(a)
y = 1.4486x - 2.2907
R^2 = 0.824
FCF (t CO_2eq ha^{-1})
CF-CO_2 (t CO_2eq ha^{-1})
(b)
y = 3.4563x - 2.2314
R^2 = 0.8292
FCF (t CO_2eq ha^{-1})
CF-N_2O (t CO_2eq ha^{-1})
(c)
y = -0.6547x + 2.8315
R^2 = 0.0434
FCF (t CO_2eq ha^{-1})
OF-CO_2 (t CO_2eq ha^{-1})
(d)
y = -144.34x + 2.998
R^2 = 0.0852
FCF (t CO_2eq ha^{-1})
OF-N_2O (t CO_2eq ha^{-1})

Fig.S2 Correlation analysis of FCF and CF-CO_2(a), FCF and CF-N_2O (b), FCF and OF-CO_2 (c) and FCF and OF-N_2O (d).

Note: The farm CF (FCF) expressed in terms of net GHG emissions per unit orchard area. CF-CO_2, CF-N_2O, OF-CO_2 and OF-N_2O denote CO_2 from chemical fertilizer production, N_2O from chemical fertilizer application, CO_2 from organic fertilizer production and N_2O from organic fertilizer application, respectively.

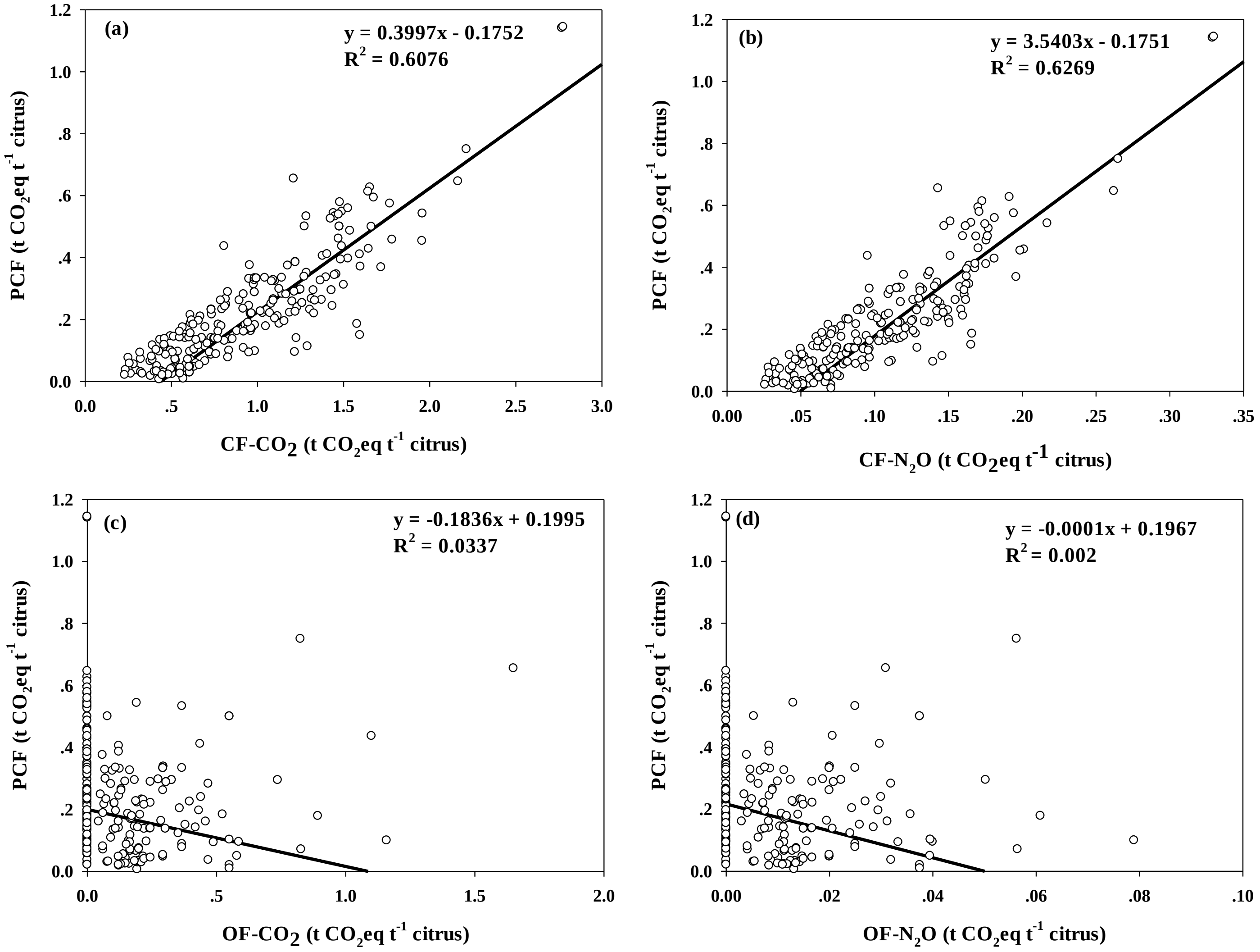

(a)
y = 0.3997x - 0.1752
R^2 = 0.6076
PCF (t CO_2eq t^{-1} citrus)
CF-CO_2 (t CO_2eq t^{-1} citrus)
(b)
y = 3.5403x - 0.1751
R^2 = 0.6269
PCF (t CO_2eq t^{-1} citrus)
CF-N_2O (t CO_2eq t^{-1} citrus)
(c)
y = -0.1836x + 0.1995
R^2 = 0.0337
PCF (t CO_2eq t^{-1} citrus)
OF-CO_2 (t CO_2eq t^{-1} citrus)
(d)
y = -0.0001x + 0.1967
R^2 = 0.002
PCF (t CO_2eq t^{-1} citrus)
OF-N_2O (t CO_2eq t^{-1} citrus)

Fig.S3 Correlation analysis of PCF and CF-CO_2(a), PCF and CF-N_2O (b), PCF and OF-CO_2 (c) and PCF and OF-N_2O (d).

Note: The product CF (PCF) expressed in terms of net GHG emissions per unit of fresh citrus yield. CF-CO_2, CF-N_2O, OF-CO_2 and OF-N_2O denote CO_2 from chemical fertilizer production, N_2O from chemical fertilizer application, CO_2 from organic fertilizer production and N_2O from organic fertilizer application.
